# Comprehensive structural study of lanthanide(III) chloride hydrates: [*RE*Cl_3_·*x*H_2_O (*RE* = La–Nd, Sm–Lu; *x* = 6, 7)]

**DOI:** 10.1107/S2056989024011319

**Published:** 2024-11-28

**Authors:** Thimira Kandabadage, Beau Legnon, Sviatoslav Baranets

**Affiliations:** a Department of Chemistry Louisiana State University,Baton Rouge LA 70803 USA; b Episcopal School of Baton Rouge, 3200 Woodland Ridge Blvd, Baton Rouge, LA 70816, USA; University of Aberdeen, United Kingdom

**Keywords:** crystal structure, rare-earth metals, periodic trends, coordination, hydrates

## Abstract

A comprehensive crystallographic study is presented of the complete series of rare-earth(III) chloride hydrates. Early lanthanides form dimeric [(H_2_O)_7_*RE*(μ-Cl)_2_*RE*(H_2_O)_7_]^4+^ binuclear complexes in which each *RE* atom (*RE* = La^3+^, Ce^3+^) is coordinated by seven H_2_O mol­ecules and two bridging inner-sphere chloride ions. Di-μ-chlorido-bis­[hepta­aqua­lanthanide(III)] tetra­chloride, [(H_2_O)_7_*RE*(μ-Cl)_2_*RE*(H_2_O)_7_]Cl_4_, crystallizes in the triclinic space group *P*1. Heavier lanthanides exhibit monomeric [*RE*Cl_2_(H_2_O)_6_]^+^ units where each *RE* atom (*RE* = Pr^3+^, Nd^3+^, Sm^3+^–Lu^3+^) is coordinated by six H_2_O mol­ecules and two inner-sphere chloride ions. Hexa­aqua­dichlorido­lanthanide(III) chlorides, [*RE*Cl_2_(H_2_O)_6_]Cl, adopt the monoclinic space group *P*2/*c.* In both structures, the cationic *inner-sphere* complex is counter-charged by the corresponding number of *outer-sphere* Cl^−^ anions, in which the metal ion and outer-sphere chloride ion lie on crystallographic twofold axes. Crystal structures for all compounds were determined in high quality with refined H-atom positions and were collected at the same temperature (100 K), providing a uniform structural dataset and addressing discrepancies in previous reports. The crystal structure of [HoCl_2_(H_2_O)_6_]Cl is reported for the first time.

## Abstract 2

This study presents a comprehensive crystallographic analysis of the lanthanide(III) chloride hydrates [*RE*Cl_3_·xH_2_O (*RE* = La–Nd, Sm–Lu; *x* = 6, 7)], offering new structural data for the series. The research highlights the influence of hydration levels and lanthanide contraction on the crystal structures, while identifying missing data for specific hydrates in existing structural databases.

## 1. Chemical context and database survey

The rare-earth trivalent metal chloride hydrates [*RE*Cl_3_·*x*H_2_O (*RE* = La–Nd, Sm–Lu; *x* = 6, 7)] are commonly used as precursors in the synthesis of complex inorganic and organometallic compounds (Boyle & Steele, 2011 ▸). Most lanthanides are characterized by the presence of partially filled 4*f* orbitals, which significantly influence the chemical and physical properties of these elements and their compounds, offering a broad landscape for potential applications. For instance, lanthanide chloride salts possess catalytic (Narasimhulu *et al.*, 2007 ▸), luminescent (Hsieh *et al.*, 2013 ▸), scintillation (Boatner *et al.*, 2013 ▸), and magnetic properties (Layfield & Murugesu, 2015 ▸), to name a few. The compositional and structural aspects of the lanthanide (III) chloride hydrate chemistry are well established, with nearly the entire series of *RE*Cl_3_·*x*H_2_O compositions identified (Boyle *et al.*, 2010 ▸; Cotton & Harrowfield, 2011 ▸). However, our database survey indicates that while most of the compounds presented in this work are listed in the ICSD database (Release 2024.1; Zagorac *et al.*, 2019 ▸), structural data for HoCl_3_·6H_2_O and TmCl_3_·6H_2_O are missing from the ICSD, and for HoCl_3_·6H_2_O, from the CSD (Release 2024.2; Groom *et al.*, 2016 ▸) databases (Bakakin *et al.*, 1975 ▸; Bel’skii & Struchkov, 1965 ▸; Boyle *et al.*, 2010 ▸; Habenschuss & Spedding, 1979 ▸, 1980*a* ▸,*b* ▸,*c* ▸,*d* ▸, 1978 ▸; Hsieh *et al.*, 2013 ▸; Kepert *et al.*, 1983 ▸; Knopf *et al.*, 2015 ▸; Levason & Webster, 2002 ▸; Louer *et al.*, 1989 ▸; Marezio *et al.*, 1961 ▸; Narasimhulu *et al.*, 2007 ▸; Peterson *et al.*, 1979 ▸; Reuter *et al.*, 1994 ▸; Reuter & Frenzen, 1994 ▸; Rheingold & King, 1989 ▸; Tambornino *et al.*, 2014 ▸; Wegner *et al.*, 2018 ▸; Chen *et al.*, 1991 ▸).
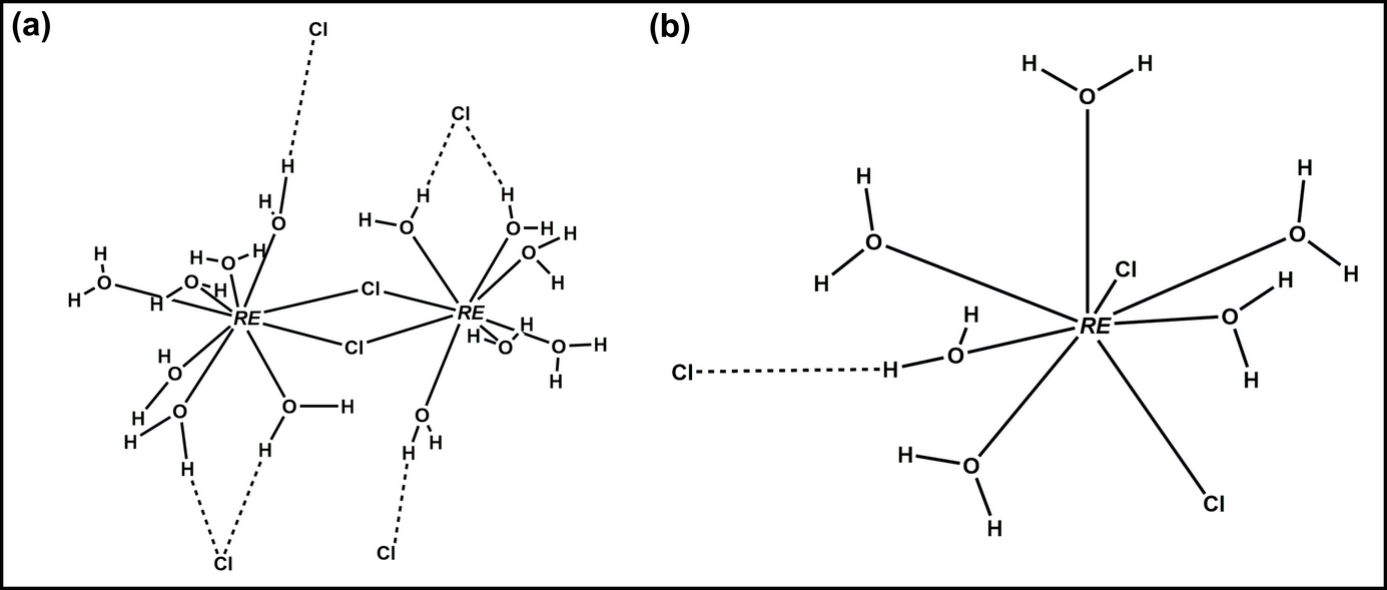


In addition, most of the reported datasets are of standard quality, lacking refined hydrogen atoms, which complicates the unambiguous inter­pretation of hydrogen bonding and the crystal packing. Another inconsistency involves data collection temperatures, which vary across the series in the previous reports. This study presents a comprehensive crystallographic analysis by providing a uniform structural dataset for the complete series of rare-earth metal(III) chloride hydrates except for radioactive promethium.

## 2. Structural commentary

The rare-earth metal(III) chloride hydrates [*RE*Cl_3_·*x*H_2_O (*RE* = La–Nd, Sm–Lu; *x* = 6, 7)] adopt two different structure types, with the rare-earth metal being coordinated by seven (for *RE* = La, Ce) or six (for *RE* = Pr, Nd, Sm–Lu) water mol­ecules. The degree of hydration is likely influenced by steric effects and aligns well with the decreasing trend of the atomic radii within the lanthanide series (Fig. 5). Metal–ligand bond distances for the La and Ce phases are listed in Tables 1 ▸ and 2 ▸, respectively, and the metal–ligand distances for the hexa­hydrates are summarized in Table 3 ▸. The hydrogen-bond geometrical data for the complete series (La–Lu) are listed in Table 4 ▸ ▸ ▸ ▸ ▸ ▸ ▸ ▸ ▸ ▸ ▸ ▸ ▸ to 17 ▸.

**Figure 1:**
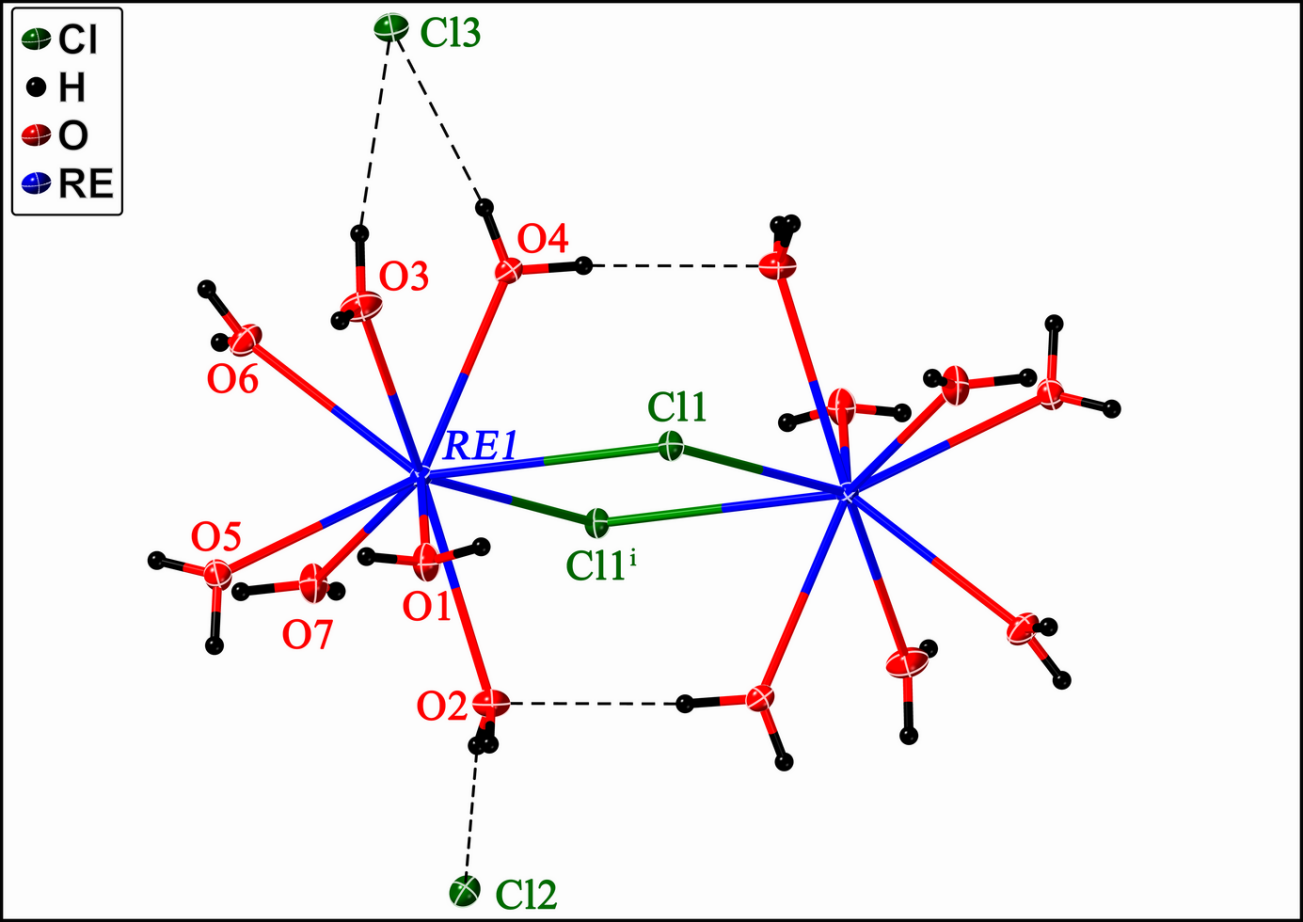
Mol­ecular structure of the dimeric [(H_2_O)_7_*RE*(μ-Cl)_2_*RE*(H_2_O)_7_]Cl_4_ compound (*RE* = La, Ce) with displacement ellipsoids drawn at 50% probability. Hydrogen bonds are shown as bold dashed lines. *RE*, Cl, O, and H atoms are represented in blue, green, red, and black, respectively. Symmetry code: (i) −*x*, −*y*, 1 − *z*.

The early rare-earth metal (*RE* = La–Pr) chloride hydrates adopt the [LaCl_3_(H_2_O)_7_] structure type and crystallize in the triclinic space group *P*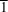, although there are a few reports on *RE*Cl_3_(H_2_O)_3_ trihydrates (*RE* = La, Ce) crystallizing in the hexa­gonal space group *P*
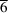
2*m* or the ortho­rhom­bic space group *Pnma* (Reuter *et al.*, 1994 ▸; Reuter & Frenzen, 1994 ▸). This structure type is characterized by the Wyckoff sequence *i*^11^ (excluding H atoms) and the Pearson Symbol *aP*50, with 11 atomic sites occupying general positions. The crystal structure of [*RE*Cl_3_(H_2_O)_7_] (*RE* = La, Ce, Pr) is better described as a dimeric [(H_2_O)_7_*RE*(μ-Cl)_2_*RE*(H_2_O)_7_]Cl_4_ mol­ecule in which each *RE* atom is coordinated by seven water mol­ecules and the two lanthanide ions are linked by the two *inner-sphere* bridging chloride (μ-Cl) anions (Fig. 1 ▸). This complex [(H_2_O)_7_*RE*(μ-Cl)_2_*RE*(H_2_O)_7_]^4+^*inner-sphere* cation is charge-balanced by four Cl^−^ anions located in the *outer-sphere*. While we identified the La- and Ce-bearing analogs, we did not find a Pr-containing compound within this structure type.

**Figure 2:**
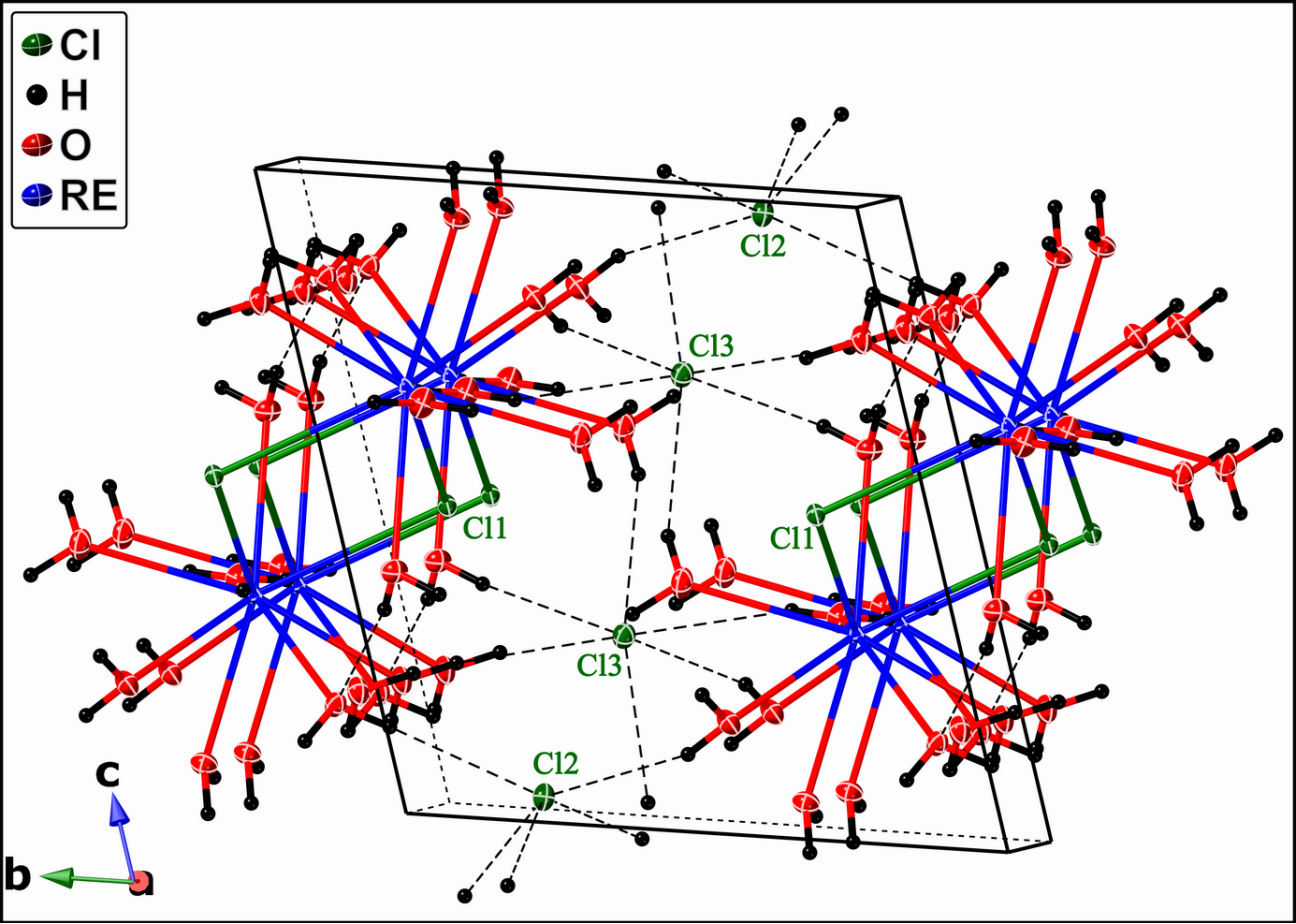
Schematic view of the packing diagram for the dimeric [(H_2_O)_7_*RE*(μ-Cl)_2_*RE*(H_2_O)_7_]Cl_4_ compound (*RE* = La, Ce). Hydrogen bonds are shown as bold dashed lines, and the unit cell is outlined. *RE*, Cl, O, and H atoms are represented in blue, green, red, and black, respectively.

Each unit cell consists of a single [(H_2_O)_7_*RE*(μ-Cl)_2_*RE*(H_2_O)_7_]Cl_4_ (*RE* = La, Ce) formula unit with [(H_2_O)_7_*RE*(μ-Cl)_2_*RE*(H_2_O)_7_]^4+^ binuclear cations linked *via* O—H⋯Cl hydrogen bonds ranging in H⋯Cl length from *ca*. 2.25 Å to 2.51 Å. Two outer-sphere Cl atoms link neighboring cations *via* these hydrogen bonds (Fig. 2 ▸).

**Figure 3:**
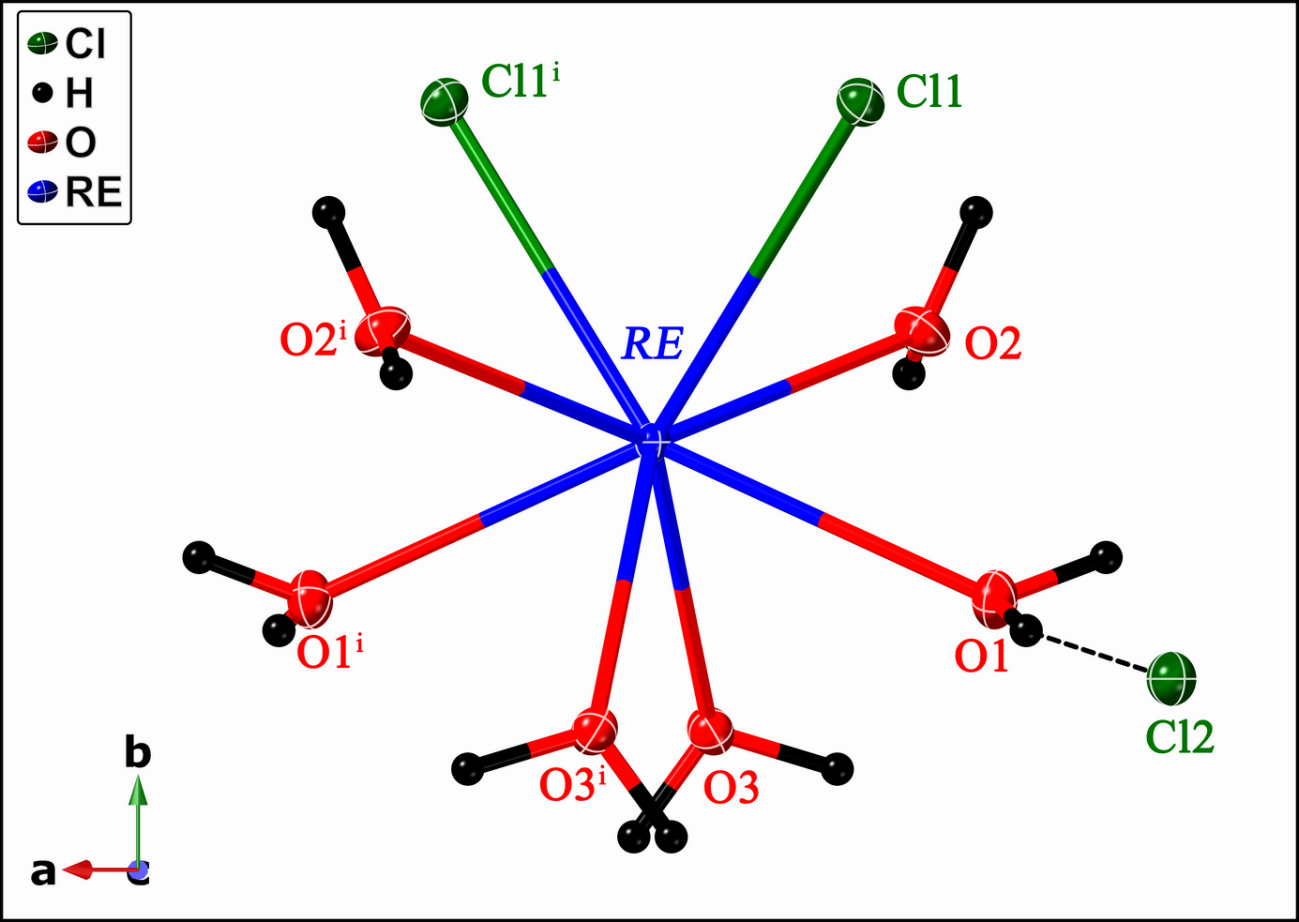
Mol­ecular structure of the *RE*Cl_2_(H_2_O)_6_Cl compound (*RE* = Pr, Nd, Sm–Lu) with displacement ellipsoids drawn at 50% probability. Hydrogen bonds are shown as bold dashed lines. *RE*, Cl, O, and H atoms are represented in blue, green, red, and black, respectively. Symmetry code: (i) 1 − *x*, *y*, 1/2 - *z.*

The heavier lanthanides (*RE* = Pr, Nd, Sm–Lu) crystallize in the monoclinic crystal system with the space group *P*2/*c* (note that most of the datasets were previously reported in the unconventional space group *P*2/*n*). They adopt the GdCl_3_·6H_2_O structure type, characterized by the Wyckoff sequence *g*^4^*fe* (excluding H atoms) and Pearson Symbol *mP*44. Each *RE*Cl_3_·6H_2_O formula unit consists of an [*RE*Cl_2_(H_2_O)_6_]^+^*inner-sphere* monomeric cation charge-balanced by a Cl^−^ anion located in the *outer-sphere* (Fig. 3 ▸). The *RE*^3+^ cation is coordinated by six water mol­ecules and two chloride anions in distorted square anti­prism fashion and is positioned on a crystallographic twofold axis.

**Figure 4:**
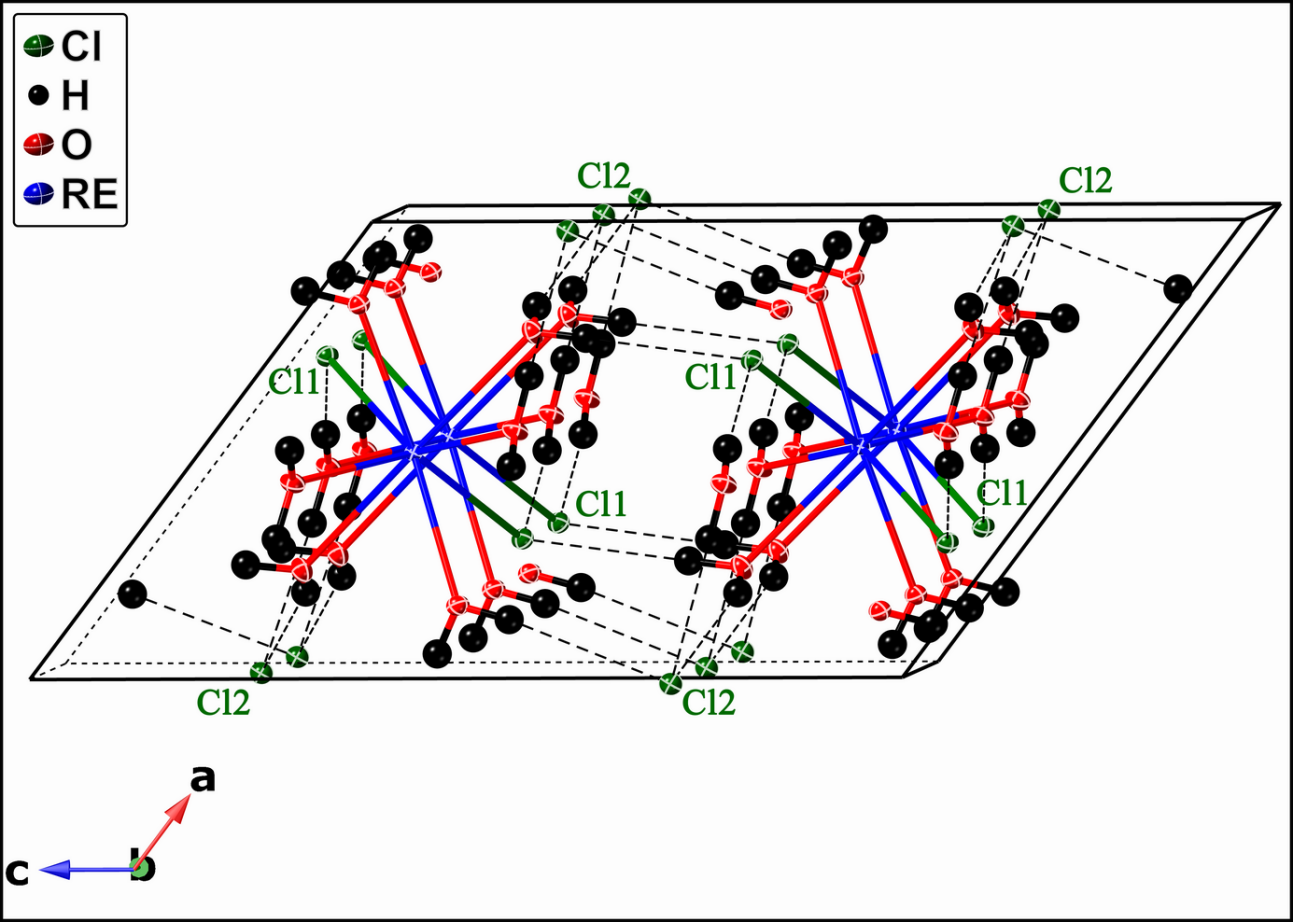
Schematic view of the packing diagram for the *RE*Cl_2_(H_2_O)_6_Cl compound (*RE* = Pr, Nd, Sm–Lu). Hydrogen bonds are shown as bold dashed lines, and the unit cell is outlined. *RE*, Cl, O, and H atoms are represented in blue, green, red, and black, respectively.

Each unit cell of the *RE*Cl_2_(H_2_O)_6_Cl (*RE* = Pr, Nd, Sm–Lu) structure type contains two formula units, with [*RE*Cl_2_(H_2_O)_6_]^+^ cations linked *via* O—H⋯Cl and O—H⋯O hydrogen bonds (Fig. 4 ▸). Each outer-sphere chloride ion accepts six O—H⋯Cl contacts (*ca*. 2.36–2.48 Å for the Ho-bearing structure), thus linking six neighboring cations. The inner sphere Cl atom forms three hydrogen O—H⋯Cl bonds (*ca*. 2.35–2.40 Å for the Ho-bearing structure), thus linking four [*RE*Cl_2_(H_2_O)_6_]^+^ cations.

**Figure 5:**
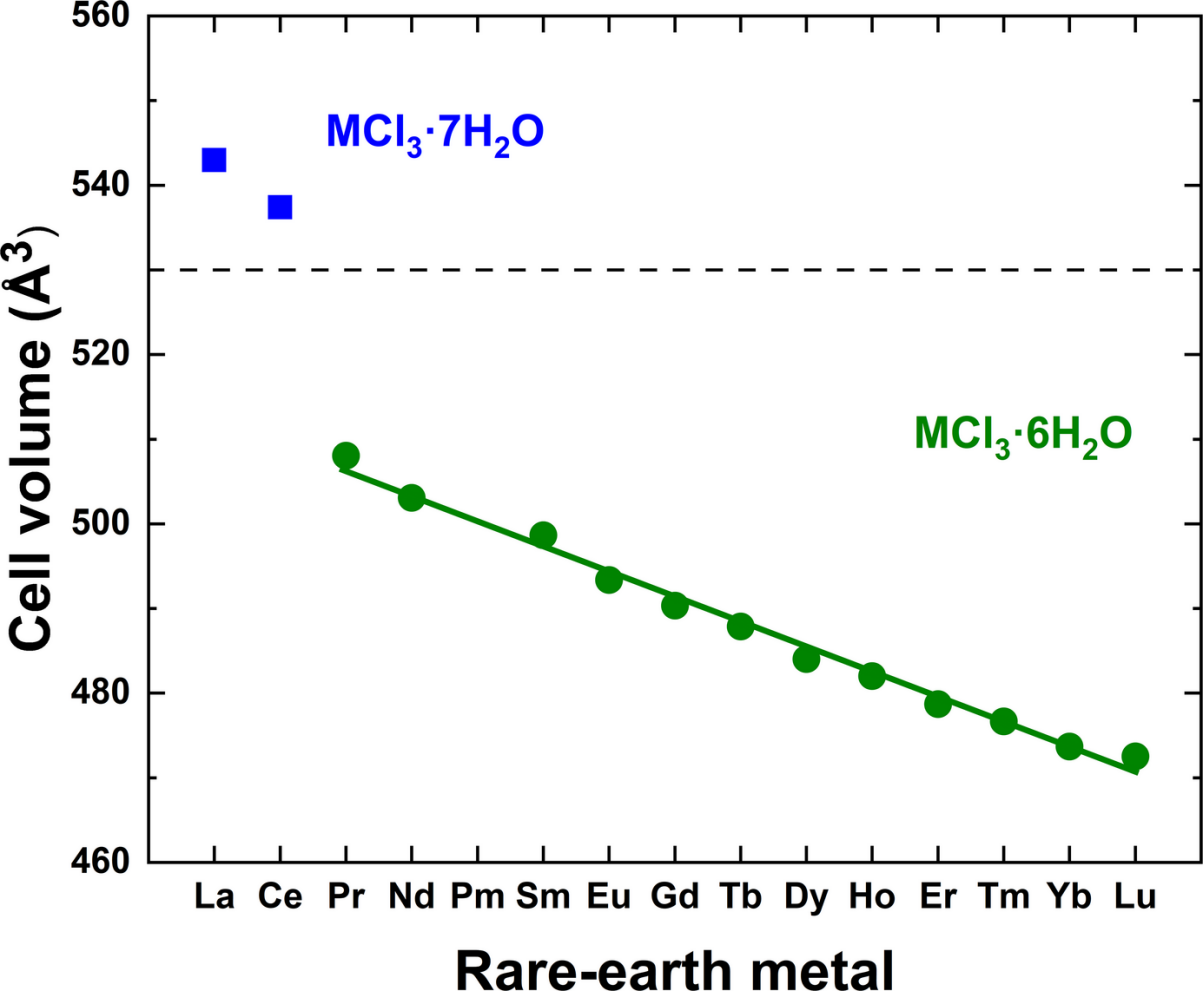
Variation of unit-cell volumes for *RE*Cl_3_·*x*H_2_O (*RE* = La–Nd, Sm–Lu; *x* = 6, 7) as determined from single-crystal X-ray diffraction experiments.

**Figure 6:**
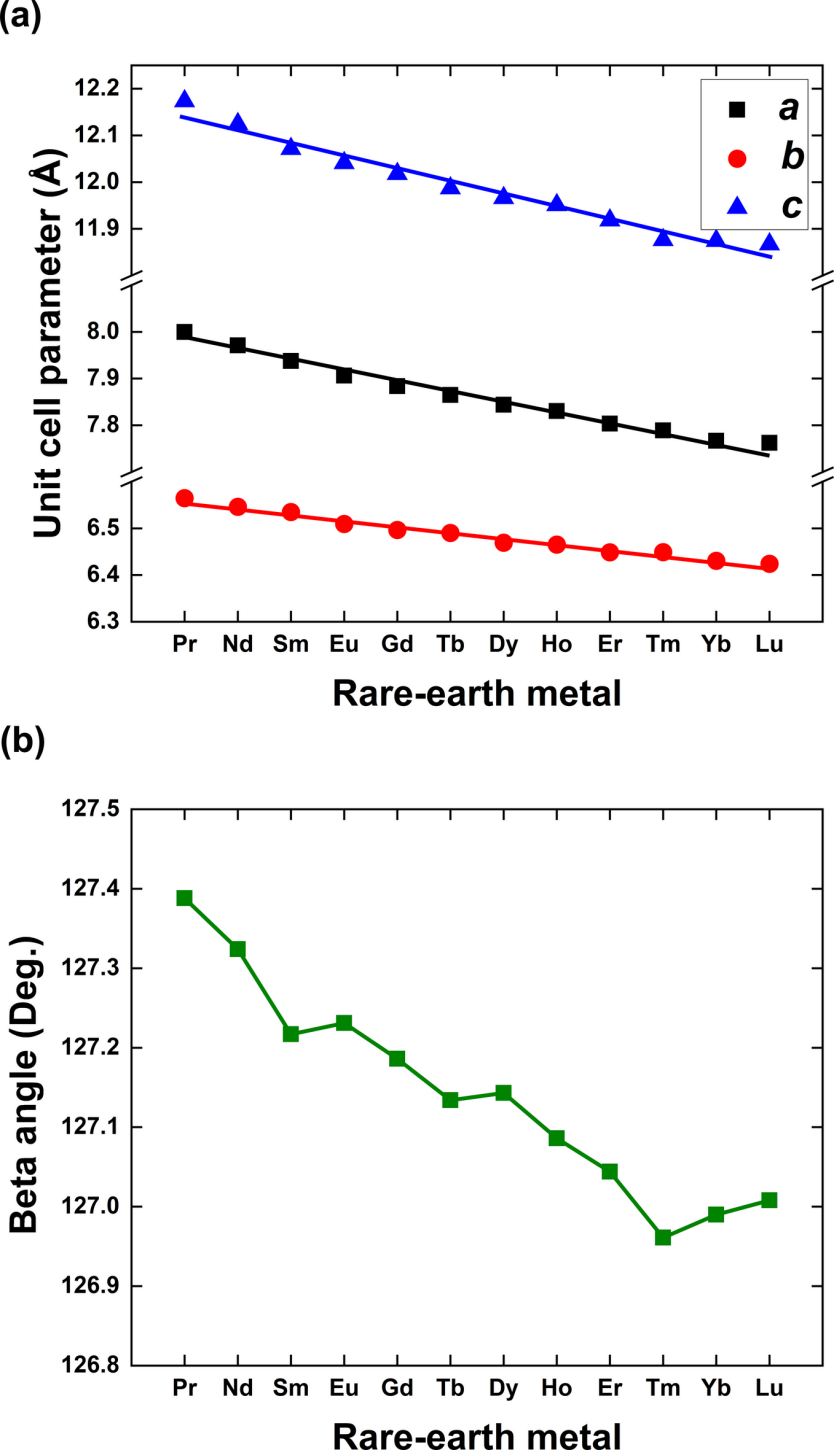
Variation of unit-cell parameters for *RE*Cl_3_·6H_2_O as determined from single-crystal X-ray diffraction experiments.

An analysis of unit-cell volumes across the La–Lu series indicates a gradual decrease (Fig. 5 ▸), which is expected due to the decreasing trend for atomic radii of the lanthanides. This reduction in atomic size for heavier *RE* leads to smaller unit cell volumes in their crystal structures. The notably larger unit-cell volumes of the La- and Ce-bearing compounds are explained by the higher number of coordinated water mol­ecules and different structural arrangements. The variations of other parameters are shown in Fig. 6 ▸.

## 3. Synthesis and crystallization

Single-crystal X-ray diffraction (SCXRD) data collections were performed using commercially available chemicals supplied by Edgetech Industries LLC without further purification. However, large single crystals suitable for property measurements can also be obtained by recrystallization, as reported elsewhere (Chen *et al.*, 1991 ▸, Peterson *et al.*, 1979 ▸).

## 4. Refinement and Methodology

Crystal data are summarized in Table 18 ▸. SCXRD data were collected under a constant stream of cold nitro­gen gas to protect the crystals from air and moisture and to control the measurement temperature. After the structures were solved, the *STRUCTURE TIDY* (Gelato & Parthé, 1987 ▸) program was used to standardize the atomic coordinates, and the unit cell was transformed to the conventional monoclinic space group *P*2/*c* for the *RE*Cl_2_(H_2_O)_6_Cl series.

## Supplementary Material

Crystal structure: contains datablock(s) La, Ce, Pr, Nd, Sm, Eu, Gd, Tb, Dy, Ho, Er, Tm, Yb, Lu, Global. DOI: 10.1107/S2056989024011319/hb8106sup1.cif

CCDC references: 2404380, 2404379, 2404378, 2404377, 2404376, 2404375, 2404374, 2404373, 2404372, 2404371, 2404370, 2404369, 2404368, 2404367

Additional supporting information:  crystallographic information; 3D view; checkCIF report

## Figures and Tables

**Table 1:** Selected bond lengths (Å) for La[Chem scheme1]

La1—Cl1	2.9187 (5)	La1—O4	2.5873 (13)
La1—O1	2.5443 (13)	La1—O5	2.5233 (14)
La1—O2	2.5504 (13)	La1—O6	2.5413 (15)
La1—O3	2.5315 (13)	La1—O7	2.5643 (13)

**Table 2:** Selected bond lengths (Å) for Ce[Chem scheme1]

Ce1—Cl1	2.8986 (2)	Ce1—O4	2.5580 (7)
Ce1—O1	2.5276 (7)	Ce1—O5	2.5018 (7)
Ce1—O2	2.5229 (7)	Ce1—O6	2.5214 (7)
Ce1—O3	2.5076 (8)	Ce1—O7	2.5397 (8)

**Table 3:** Bond distances (Å) for hexa­hydrates

*RE*	Cl1	O1	O2	O3
Pr	2.8314 (4)	2.4677 (13)	2.4818 (13)	2.4513 (12)
Nd	2.8145 (2)	2.4532 (7)	2.4629 (6)	2.4328 (7)
Sm	2.7906 (3)	2.4269 (8)	2.4349 (8)	2.4057 (8)
Eu	2.7788 (2)	2.4131 (7)	2.4206 (7)	2.3884 (7)
Gd	2.7699 (3)	2.4026 (9)	2.4101 (8)	2.3762 (8)
Tb	2.7617 (3)	2.3870 (9)	2.3940 (9)	2.3646 (9)
Dy	2.7500 (2)	2.3735 (8)	2.3795 (8)	2.3480 (8)
Ho	2.7425 (3)	2.3646 (10)	2.3705 (9)	2.3372 (9)
Er	2.7309 (3)	2.3538 (9)	2.3578 (9)	2.3239 (9)
Tm	2.7246 (2)	2.3414 (7)	2.3446 (8)	2.3142 (7)
Yb	2.7173 (3)	2.3293 (8)	2.3328 (8)	2.3003 (8)
Lu	2.7113 (4)	2.3246 (11)	2.3272 (12)	2.2917 (12)

**Table 4:** Hydrogen-bond geometry (Å, °) for La[Chem scheme1]

*D*—H⋯*A*	*D*—H	H⋯*A*	*D*⋯*A*	*D*—H⋯*A*
O1—H1*A*⋯Cl3^i^	0.75 (3)	2.48 (3)	3.1947 (15)	161 (3)
O1—H1*B*⋯Cl2	0.79 (3)	2.38 (3)	3.1365 (16)	161 (2)
O2—H2*A*⋯Cl2^ii^	0.80 (3)	2.38 (3)	3.1378 (14)	157 (3)
O2—H2*B*⋯Cl2^iii^	0.79 (3)	2.26 (3)	3.0382 (13)	172 (3)
O3—H3*A*⋯Cl3	0.82 (3)	2.47 (3)	3.2552 (15)	160 (3)
O3—H3*B*⋯Cl1^i^	0.78 (3)	2.92 (3)	3.2954 (13)	112 (2)
O3—H3*B*⋯Cl2	0.78 (3)	2.72 (3)	3.4160 (16)	150 (3)
O4—H4*A*⋯O2^iv^	0.78 (3)	2.08 (3)	2.859 (2)	174 (3)
O4—H4*B*⋯Cl3	0.84 (3)	2.31 (3)	3.1488 (15)	179 (2)
O5—H5*A*⋯Cl3^v^	0.80 (3)	2.34 (3)	3.1308 (14)	167 (3)
O5—H5*B*⋯Cl2^vi^	0.75 (3)	2.44 (3)	3.1690 (15)	163 (3)
O5—H5*B*⋯O5^vi^	0.75 (3)	2.67 (3)	2.986 (3)	108 (2)
O6—H6*A*⋯Cl3^vii^	0.77 (3)	2.57 (3)	3.3288 (16)	168 (3)
O6—H6*B*⋯Cl1^viii^	0.80 (3)	2.93 (3)	3.3705 (14)	117 (2)
O6—H6*B*⋯O4^ix^	0.80 (3)	2.24 (3)	3.038 (2)	169 (3)

Symmetry codes: (i) 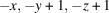; (ii) 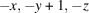; (iii) 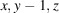; (iv) 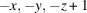; (v) 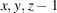; (vi) 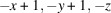; (vii) 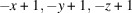; (viii) 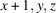; (ix) 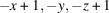.

**Table 5:** Hydrogen-bond geometry (Å, °) for Ce[Chem scheme1]

*D*—H⋯*A*	*D*—H	H⋯*A*	*D*⋯*A*	*D*—H⋯*A*
O1—H1*A*⋯Cl3^i^	0.762 (19)	2.458 (19)	3.1945 (8)	162.9 (18)
O1—H1*B*⋯Cl2	0.77 (2)	2.405 (19)	3.1380 (8)	160.6 (17)
O2—H2*A*⋯Cl2^ii^	0.797 (19)	2.251 (19)	3.0368 (8)	168.6 (17)
O2—H2*B*⋯Cl2^iii^	0.783 (19)	2.411 (19)	3.1383 (8)	155.0 (16)
O3—H3*A*⋯Cl3	0.79 (2)	2.50 (2)	3.2501 (9)	160.9 (18)
O3—H3*B*⋯Cl1^i^	0.80 (2)	2.91 (2)	3.3040 (8)	112.9 (16)
O3—H3*B*⋯Cl2	0.80 (2)	2.71 (2)	3.4188 (9)	147.3 (18)
O4—H4*A*⋯O2^iv^	0.800 (17)	2.068 (17)	2.8609 (11)	171.2 (16)
O4—H4*B*⋯Cl3	0.781 (18)	2.369 (18)	3.1466 (8)	173.7 (16)
O5—H5*A*⋯Cl3^v^	0.798 (17)	2.354 (17)	3.1286 (8)	164.0 (16)
O5—H5*B*⋯Cl2^vi^	0.768 (19)	2.446 (19)	3.1699 (8)	157.6 (17)
O5—H5*B*⋯O5^vi^	0.768 (19)	2.601 (17)	2.9858 (15)	112.9 (15)
O6—H6*A*⋯Cl1^vii^	0.82 (2)	2.936 (18)	3.3662 (8)	115.2 (14)
O6—H6*A*⋯O4^viii^	0.82 (2)	2.25 (2)	3.0451 (11)	164.9 (17)
O6—H6*B*⋯Cl3^ix^	0.79 (2)	2.55 (2)	3.3165 (8)	164.4 (18)

Symmetry codes: (i) 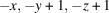; (ii) 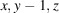; (iii) 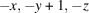; (iv) 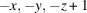; (v) 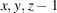; (vi) 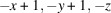; (vii) 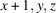; (viii) 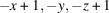; (ix) 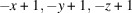.

**Table 6:** Hydrogen-bond geometry (Å, °) for Pr[Chem scheme1]

*D*—H⋯*A*	*D*—H	H⋯*A*	*D*⋯*A*	*D*—H⋯*A*
O1—H1*A*⋯Cl1^i^	0.75 (3)	2.42 (3)	3.1498 (14)	166 (3)
O1—H1*B*⋯Cl2^ii^	0.78 (3)	2.40 (3)	3.1706 (13)	172 (2)
O2—H2*A*⋯Cl1^iii^	0.76 (3)	2.40 (3)	3.1575 (13)	175 (2)
O2—H2*B*⋯Cl2^iv^	0.77 (2)	2.49 (2)	3.2291 (14)	163 (2)
O3—H3*A*⋯Cl1^v^	0.78 (3)	2.36 (3)	3.1303 (14)	174 (2)
O3—H3*B*⋯Cl2	0.80 (3)	2.41 (3)	3.1919 (14)	166 (2)

Symmetry codes: (i) 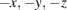; (ii) 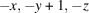; (iii) 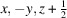; (iv) 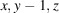; (v) 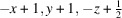.

**Table 7:** Hydrogen-bond geometry (Å, °) for Nd[Chem scheme1]

*D*—H⋯*A*	*D*—H	H⋯*A*	*D*⋯*A*	*D*—H⋯*A*
O1—H1*A*⋯Cl1^i^	0.749 (16)	2.418 (16)	3.1491 (7)	165.6 (16)
O1—H1*B*⋯Cl2^ii^	0.722 (17)	2.451 (18)	3.1661 (8)	170.8 (15)
O2—H2*A*⋯Cl1^iii^	0.770 (18)	2.387 (18)	3.1552 (8)	175.3 (17)
O2—H2*B*⋯Cl2^iv^	0.760 (17)	2.490 (17)	3.2267 (8)	164.0 (15)
O3—H3*A*⋯Cl1^v^	0.793 (16)	2.337 (17)	3.1290 (7)	176.1 (15)
O3—H3*B*⋯Cl2	0.782 (16)	2.413 (16)	3.1894 (7)	172.0 (15)

Symmetry codes: (i) 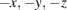; (ii) 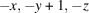; (iii) 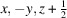; (iv) 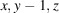; (v) 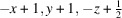.

**Table 8:** Hydrogen-bond geometry (Å, °) for Sm[Chem scheme1]

*D*—H⋯*A*	*D*—H	H⋯*A*	*D*⋯*A*	*D*—H⋯*A*
O1—H1*A*⋯Cl1^i^	0.693 (19)	2.484 (19)	3.1594 (10)	165 (2)
O1—H1*B*⋯Cl2^ii^	0.79 (3)	2.39 (3)	3.1685 (9)	169.2 (19)
O2—H2*A*⋯Cl1^iii^	0.81 (2)	2.35 (2)	3.1586 (9)	175.9 (18)
O2—H2*B*⋯Cl2^iv^	0.78 (2)	2.48 (2)	3.2358 (10)	163.6 (17)
O3—H3*A*⋯Cl1^v^	0.78 (2)	2.36 (2)	3.1367 (10)	176 (2)
O3—H3*B*⋯Cl2	0.794 (19)	2.41 (2)	3.1908 (9)	167.7 (18)

Symmetry codes: (i) 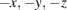; (ii) 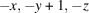; (iii) 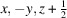; (iv) 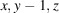; (v) 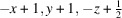.

**Table 9:** Hydrogen-bond geometry (Å, °) for Eu[Chem scheme1]

*D*—H⋯*A*	*D*—H	H⋯*A*	*D*⋯*A*	*D*—H⋯*A*
O1—H1*A*⋯Cl1^i^	0.760 (15)	2.407 (15)	3.1537 (7)	167.5 (16)
O1—H1*B*⋯Cl2^ii^	0.766 (19)	2.407 (19)	3.1649 (8)	170.6 (14)
O2—H2*A*⋯Cl1^iii^	0.784 (16)	2.373 (17)	3.1549 (8)	175.1 (14)
O2—H2*B*⋯Cl2^iv^	0.759 (16)	2.500 (15)	3.2308 (7)	162.1 (14)
O3—H3*A*⋯Cl1^v^	0.783 (16)	2.349 (16)	3.1307 (7)	175.7 (14)
O3—H3*B*⋯Cl2	0.737 (15)	2.455 (15)	3.1842 (7)	170.3 (15)

Symmetry codes: (i) 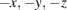; (ii) 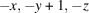; (iii) 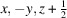; (iv) 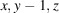; (v) 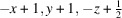.

**Table 10:** Hydrogen-bond geometry (Å, °) for Gd[Chem scheme1]

*D*—H⋯*A*	*D*—H	H⋯*A*	*D*⋯*A*	*D*—H⋯*A*
O1—H1*A*⋯Cl1^i^	0.79 (2)	2.39 (2)	3.1527 (9)	165 (2)
O1—H1*B*⋯Cl2^ii^	0.75 (2)	2.43 (2)	3.1651 (9)	170.1 (19)
O2—H2*A*⋯Cl1^iii^	0.80 (2)	2.36 (2)	3.1519 (9)	175 (2)
O2—H2*B*⋯Cl2^iv^	0.82 (2)	2.44 (2)	3.2284 (9)	161.7 (18)
O3—H3*A*⋯Cl1^v^	0.83 (2)	2.30 (2)	3.1290 (9)	178 (2)
O3—H3*B*⋯Cl2	0.79 (2)	2.40 (2)	3.1790 (9)	169.1 (19)

Symmetry codes: (i) 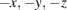; (ii) 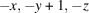; (iii) 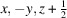; (iv) 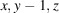; (v) 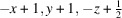.

**Table 11:** Hydrogen-bond geometry (Å, °) for Tb[Chem scheme1]

*D*—H⋯*A*	*D*—H	H⋯*A*	*D*⋯*A*	*D*—H⋯*A*
O1—H1*A*⋯Cl1^i^	0.77 (2)	2.41 (2)	3.1527 (9)	164 (2)
O1—H1*B*⋯Cl2^ii^	0.77 (2)	2.40 (2)	3.1661 (10)	172 (2)
O2—H2*A*⋯Cl1^iii^	0.82 (2)	2.33 (2)	3.1524 (10)	175 (2)
O2—H2*B*⋯Cl2^iv^	0.76 (2)	2.51 (2)	3.2283 (10)	158.8 (19)
O3—H3*A*⋯Cl1^v^	0.81 (2)	2.33 (2)	3.1297 (10)	173.2 (19)
O3—H3*B*⋯Cl2	0.77 (2)	2.43 (2)	3.1798 (9)	166 (2)

Symmetry codes: (i) 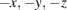; (ii) 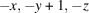; (iii) 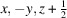; (iv) 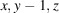; (v) 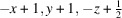.

**Table 12:** Hydrogen-bond geometry (Å, °) for Dy[Chem scheme1]

*D*—H⋯*A*	*D*—H	H⋯*A*	*D*⋯*A*	*D*—H⋯*A*
O1—H1*A*⋯Cl1^i^	0.755 (19)	2.413 (19)	3.1545 (9)	167.6 (19)
O1—H1*B*⋯Cl2^ii^	0.78 (2)	2.39 (2)	3.1655 (8)	172.6 (16)
O2—H2*A*⋯Cl1^iii^	0.79 (2)	2.37 (2)	3.1511 (9)	175.7 (19)
O2—H2*B*⋯Cl2^iv^	0.712 (19)	2.544 (19)	3.2303 (8)	162.7 (19)
O3—H3*A*⋯Cl1^v^	0.787 (19)	2.342 (19)	3.1276 (8)	176.2 (18)
O3—H3*B*⋯Cl2	0.75 (2)	2.44 (2)	3.1766 (8)	167.1 (19)

Symmetry codes: (i) 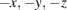; (ii) 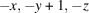; (iii) 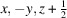; (iv) 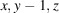; (v) 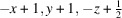.

**Table 13:** Hydrogen-bond geometry (Å, °) for Ho[Chem scheme1]

*D*—H⋯*A*	*D*—H	H⋯*A*	*D*⋯*A*	*D*—H⋯*A*
O1—H1*A*⋯Cl1^i^	0.78 (2)	2.40 (2)	3.1589 (11)	166 (2)
O1—H1*B*⋯Cl2^ii^	0.76 (2)	2.41 (2)	3.1678 (10)	170 (2)
O2—H2*A*⋯Cl1^iii^	0.79 (2)	2.37 (2)	3.1546 (10)	172 (2)
O2—H2*B*⋯Cl2^iv^	0.79 (2)	2.48 (2)	3.2320 (10)	161.6 (19)
O3—H3*A*⋯Cl1^v^	0.79 (2)	2.35 (2)	3.1309 (10)	172 (2)
O3—H3*B*⋯Cl2	0.82 (2)	2.36 (2)	3.1765 (10)	168.9 (19)

Symmetry codes: (i) 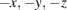; (ii) 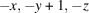; (iii) 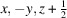; (iv) 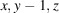; (v) 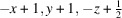.

**Table 14:** Hydrogen-bond geometry (Å, °) for Er[Chem scheme1]

*D*—H⋯*A*	*D*—H	H⋯*A*	*D*⋯*A*	*D*—H⋯*A*
O1—H1*A*⋯Cl1^i^	0.78 (2)	2.39 (2)	3.1557 (10)	166 (2)
O1—H1*B*⋯Cl2^ii^	0.76 (3)	2.41 (3)	3.1632 (10)	170 (2)
O2—H2*A*⋯Cl1^iii^	0.76 (2)	2.39 (2)	3.1502 (10)	176 (2)
O2—H2*B*⋯Cl2^iv^	0.79 (2)	2.47 (2)	3.2287 (10)	161 (2)
O3—H3*A*⋯Cl1^v^	0.77 (2)	2.36 (2)	3.1286 (10)	172 (2)
O3—H3*B*⋯Cl2	0.78 (2)	2.41 (2)	3.1689 (9)	167 (2)

Symmetry codes: (i) 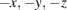; (ii) 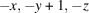; (iii) 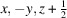; (iv) 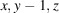; (v) 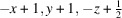.

**Table 15:** Hydrogen-bond geometry (Å, °) for Tm[Chem scheme1]

*D*—H⋯*A*	*D*—H	H⋯*A*	*D*⋯*A*	*D*—H⋯*A*
O1—H1*A*⋯Cl1^i^	0.73 (2)	2.44 (2)	3.1573 (8)	165 (2)
O1—H1*B*⋯Cl2^ii^	0.81 (2)	2.36 (2)	3.1602 (8)	169.7 (16)
O2—H2*A*⋯Cl1^iii^	0.78 (2)	2.38 (2)	3.1485 (8)	173.4 (19)
O2—H2*B*⋯Cl2^iv^	0.737 (18)	2.528 (18)	3.2301 (8)	159.7 (18)
O3—H3*A*⋯Cl1^v^	0.789 (19)	2.343 (19)	3.1280 (8)	173.4 (18)
O3—H3*B*⋯Cl2	0.75 (2)	2.43 (2)	3.1714 (8)	169.4 (17)

Symmetry codes: (i) 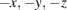; (ii) 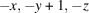; (iii) 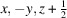; (iv) 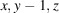; (v) 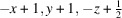.

**Table 16:** Hydrogen-bond geometry (Å, °) for Yb[Chem scheme1]

*D*—H⋯*A*	*D*—H	H⋯*A*	*D*⋯*A*	*D*—H⋯*A*
O1—H1*A*⋯Cl1^i^	0.74 (2)	2.43 (2)	3.1570 (9)	168 (2)
O1—H1*B*⋯Cl2^ii^	0.74 (2)	2.44 (2)	3.1623 (9)	167 (2)
O2—H2*A*⋯Cl1^iii^	0.72 (2)	2.44 (2)	3.1486 (9)	175 (2)
O2—H2*B*⋯Cl2^iv^	0.795 (19)	2.472 (19)	3.2287 (9)	159.5 (16)
O3—H3*A*⋯Cl1^v^	0.720 (19)	2.409 (19)	3.1273 (9)	175.4 (19)
O3—H3*B*⋯Cl2	0.84 (2)	2.33 (2)	3.1643 (8)	168.8 (17)

Symmetry codes: (i) 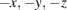; (ii) 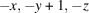; (iii) 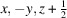; (iv) 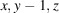; (v) 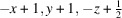.

**Table 17:** Hydrogen-bond geometry (Å, °) for Lu[Chem scheme1]

*D*—H⋯*A*	*D*—H	H⋯*A*	*D*⋯*A*	*D*—H⋯*A*
O1—H1*A*⋯Cl1^i^	0.77 (3)	2.42 (3)	3.1591 (13)	163 (3)
O1—H1*B*⋯Cl2^ii^	0.76 (3)	2.40 (3)	3.1625 (12)	171 (2)
O2—H2*A*⋯Cl1^iii^	0.73 (3)	2.42 (3)	3.1517 (13)	175 (3)
O2—H2*B*⋯Cl2^iv^	0.82 (3)	2.45 (3)	3.2327 (13)	160 (3)
O3—H3*A*⋯Cl1^v^	0.79 (3)	2.34 (3)	3.1290 (13)	174 (3)
O3—H3*B*⋯Cl2	0.77 (3)	2.41 (3)	3.1653 (12)	167 (3)

Symmetry codes: (i) 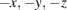; (ii) 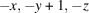; (iii) 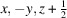; (iv) 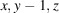; (v) 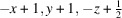.

**Table:** For all structures: *Z* = 2. Experiments were carried out at 100 K with Ag *K*α radiation, λ = 0.56086 Å using a Bruker D8 Venture Duo. Absorption was corrected for by multi-scan methods (*SADABS*; Krause *et al.*, 2016 ▸). All H-atom parameters were refined.

	[LaCl(H_2_O)_7_]Cl_2_	[CeCl(H_2_O)_7_]Cl_2_	[PrCl_2_(H_2_O)_6_]Cl
Crystal data			
*M*_r_	371.37	372.58	355.36
Crystal system, space group	Triclinic, *P* 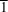	Triclinic, *P* 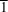	Monoclinic, *P*2/*c*
*a*, *b*, *c* (Å)	7.9432 (5), 8.2316 (4), 9.2203 (5)	7.8995 (3), 8.2129 (3), 9.1961 (3)	7.9997 (3), 6.5647 (2), 12.1734 (4)
α, β, γ (°)	70.507 (2), 73.098 (2), 81.522 (2)	70.451 (1), 73.172 (1), 81.658 (1)	90, 127.388 (1), 90
*V* (Å^3^)	542.92 (5)	537.39 (3)	507.95 (3)
μ (mm^−1^)	2.42	2.60	2.90
Crystal size (mm)	0.28 × 0.12 × 0.06	0.26 × 0.22 × 0.17	0.06 × 0.06 × 0.05

Data collection			
*T*_min_, *T*_max_	0.044, 0.064	0.214, 0.257	0.218, 0.257
No. of measured, independent and observed [*I* > 2σ(*I*)] reflections	43724, 3350, 3078	28063, 3304, 3239	15235, 1567, 1486
*R*_int_	0.069	0.031	0.058
(sin θ/λ)_max_ (Å^−1^)	0.717	0.718	0.716

Refinement			
*R*[*F*^2^ > 2σ(*F*^2^)], *wR*(*F*^2^), *S*	0.016, 0.032, 1.01	0.009, 0.019, 1.17	0.014, 0.027, 1.05
No. of reflections	3350	3304	1567
No. of parameters	157	157	71
Δρ_max_, Δρ_min_ (e Å^−3^)	0.40, −0.44	0.32, −0.30	0.38, −0.39

**Table:** 

	[NdCl_2_(H_2_O)_6_]Cl	[SmCl_2_(H_2_O)_6_]Cl	[EuCl_2_(H_2_O)_6_]Cl
Crystal data			
*M*_r_	358.69	364.80	366.41
Crystal system, space group	Monoclinic, *P*2/*c*	Monoclinic, *P*2/*c*	Monoclinic, *P*2/*c*
*a*, *b*, *c* (Å)	7.9710 (3), 6.5460 (2), 12.1246 (5)	7.9375 (8), 6.5351 (7), 12.0713 (11)	7.9062 (2), 6.5092 (2), 12.0410 (4)
α, β, γ (°)	90, 127.324 (1), 90	90, 127.217 (2), 90	90, 127.231 (1), 90
*V* (Å^3^)	503.09 (3)	498.65 (9)	493.38 (3)
μ (mm^−1^)	3.11	3.51	3.75
Crystal size (mm)	0.16 × 0.12 × 0.11	0.25 × 0.21 × 0.18	0.13 × 0.13 × 0.11

Data collection			
*T*_min_, *T*_max_	0.201, 0.257	0.204, 0.257	0.218, 0.257
No. of measured, independent and observed [*I* > 2σ(*I*)] reflections	23233, 1559, 1536	27415, 1553, 1533	21905, 1529, 1504
*R*_int_	0.036	0.046	0.038
(sin θ/λ)_max_ (Å^−1^)	0.716	0.717	0.716

Refinement			
*R*[*F*^2^ > 2σ(*F*^2^)], *wR*(*F*^2^), *S*	0.007, 0.017, 1.11	0.010, 0.023, 1.19	0.007, 0.016, 1.13
No. of reflections	1559	1553	1529
No. of parameters	72	72	72
Δρ_max_, Δρ_min_ (e Å^−3^)	0.25, −0.30	0.48, −0.85	0.29, −0.32

**Table:** 

	[GdCl_2_(H_2_O)_6_]Cl	[TbCl_2_(H_2_O)_6_]Cl	[DyCl_2_(H_2_O)_6_]Cl
Crystal data			
*M*_r_	371.70	373.37	376.95
Crystal system, space group	Monoclinic, *P*2/*c*	Monoclinic, *P*2/*c*	Monoclinic, *P*2/*c*
*a*, *b*, *c* (Å)	7.8835 (3), 6.4964 (3), 12.0176 (4)	7.8646 (3), 6.4903 (3), 11.9871 (5)	7.8439 (3), 6.4693 (3), 11.9660 (5)
α, β, γ (°)	90, 127.186 (1), 90	90, 127.134 (1), 90	90, 127.143 (1), 90
*V* (Å^3^)	490.33 (3)	487.79 (4)	484.02 (4)
μ (mm^−1^)	3.99	4.24	4.51
Crystal size (mm)	0.33 × 0.25 × 0.20	0.3 × 0.16 × 0.12	0.23 × 0.19 × 0.14

Data collection			
*T*_min_, *T*_max_	0.172, 0.257	0.223, 0.257	0.184, 0.257
No. of measured, independent and observed [*I* > 2σ(*I*)] reflections	26021, 1532, 1520	11117, 1503, 1481	21844, 1501, 1485
*R*_int_	0.037	0.024	0.037
(sin θ/λ)_max_ (Å^−1^)	0.718	0.716	0.718

Refinement			
*R*[*F*^2^ > 2σ(*F*^2^)], *wR*(*F*^2^), *S*	0.009, 0.021, 1.23	0.009, 0.020, 1.10	0.008, 0.018, 1.19
No. of reflections	1532	1503	1501
No. of parameters	72	72	72
Δρ_max_, Δρ_min_ (e Å^−3^)	0.44, −0.38	0.36, −0.37	0.42, −0.48

**Table:** 

	[HoCl_2_(H_2_O)_6_]Cl	[ErCl_2_(H_2_O)_6_]Cl	[TmCl_2_(H_2_O)_6_]Cl
Crystal data			
*M*_r_	379.38	381.71	383.38
Crystal system, space group	Monoclinic, *P*2/*c*	Monoclinic, *P*2/*c*	Monoclinic, *P*2/*c*
*a*, *b*, *c* (Å)	7.8303 (3), 6.4651 (2), 11.9509 (4)	7.8035 (2), 6.4488 (2), 11.9182 (4)	7.7889 (4), 6.4490 (3), 11.8760 (6)
α, β, γ (°)	90, 127.086 (1), 90	90, 127.044 (1), 90	90, 126.961 (1), 90
*V* (Å^3^)	482.63 (3)	478.71 (3)	476.66 (4)
μ (mm^−1^)	4.77	5.07	5.37
Crystal size (mm)	0.14 × 0.10 × 0.07	0.23 × 0.22 × 0.17	0.17 × 0.11 × 0.10

Data collection			
*T*_min_, *T*_max_	0.222, 0.257	0.175, 0.257	0.207, 0.257
No. of measured, independent and observed [*I* > 2σ(*I*)] reflections	10309, 1480, 1454	24599, 1481, 1479	16364, 1481, 1464
*R*_int_	0.026	0.034	0.033
(sin θ/λ)_max_ (Å^−1^)	0.715	0.717	0.717

Refinement			
*R*[*F*^2^ > 2σ(*F*^2^)], *wR*(*F*^2^), *S*	0.009, 0.020, 1.07	0.009, 0.020, 1.30	0.007, 0.016, 1.15
No. of reflections	1480	1481	1481
No. of parameters	72	72	72
Δρ_max_, Δρ_min_ (e Å^−3^)	0.31, −0.36	0.47, −0.88	0.39, −0.48

**Table:** 

	[YbCl_2_(H_2_O)_6_]Cl	[LuCl_2_(H_2_O)_6_]Cl
Crystal data		
*M*_r_	387.49	389.42
Crystal system, space group	Monoclinic, *P*2/*c*	Monoclinic, *P*2/*c*
*a*, *b*, *c* (Å)	7.7666 (2), 6.4305 (2), 11.8745 (3)	7.7621 (3), 6.4241 (3), 11.8671 (5)
α, β, γ (°)	90, 126.991 (1), 90	90, 127.008 (1), 90
*V* (Å^3^)	473.69 (2)	472.54 (4)
μ (mm^−1^)	5.69	6.00
Crystal size (mm)	0.30 × 0.23 × 0.20	0.17 × 0.14 × 0.14

Data collection		
*T*_min_, *T*_max_	0.168, 0.257	0.201, 0.257
No. of measured, independent and observed [*I* > 2σ(*I*)] reflections	19224, 1464, 1439	23799, 1464, 1436
*R*_int_	0.035	0.041
(sin θ/λ)_max_ (Å^−1^)	0.717	0.718

Refinement		
*R*[*F*^2^ > 2σ(*F*^2^)], *wR*(*F*^2^), *S*	0.008, 0.017, 1.19	0.009, 0.020, 1.28
No. of reflections	1464	1464
No. of parameters	72	72
Δρ_max_, Δρ_min_ (e Å^−3^)	0.62, −0.74	0.78, −0.82

Computer programs: *SAINT* (Bruker, 2016 ▸), *SHELXT2018/2* (Sheldrick, 2015*a* ▸), *SHELXL2018/1* (Sheldrick, 2015*b* ▸), *CrystalMaker* (Palmer, 2014 ▸) and *publCIF* (Westrip, 2010 ▸).

## supplementary crystallographic information

### Di-µ-chlorido-bis[heptaaqualanthanum(III)] tetrachloride (La) Crystal data

**Table:**

[LaCl(H_2_O)_7_]Cl_2_	*Z* = 2
*M**_r_* = 371.37	*F*(000) = 356
Triclinic, *P*1	*D*_x_ = 2.272 Mg m^−^^3^
*a* = 7.9432 (5) Å	Ag *K*α radiation, λ = 0.56086 Å
*b* = 8.2316 (4) Å	Cell parameters from 9869 reflections
*c* = 9.2203 (5) Å	θ = 2.4–23.7°
α = 70.507 (2)°	µ = 2.42 mm^−^^1^
β = 73.098 (2)°	*T* = 100 K
γ = 81.522 (2)°	Plate, colorless
*V* = 542.92 (5) Å^3^	0.28 × 0.12 × 0.06 mm

### Di-µ-chlorido-bis[heptaaqualanthanum(III)] tetrachloride (La) Data collection

**Table:**

Bruker D8 Venture Duo diffractometer	3078 reflections with *I* > 2σ(*I*)
ω scans	*R*_int_ = 0.069
Absorption correction: multi-scan SADABS-2016/2 (Bruker, 2016)	θ_max_ = 23.7°, θ_min_ = 2.1°
*T*_min_ = 0.044, *T*_max_ = 0.064	*h* = −11→11
43724 measured reflections	*k* = −11→11
3350 independent reflections	*l* = −13→13

### Di-µ-chlorido-bis[heptaaqualanthanum(III)] tetrachloride (La) Refinement

**Table:**

Refinement on *F*^2^	Hydrogen site location: difference Fourier map
Least-squares matrix: full	All H-atom parameters refined
*R*[*F*^2^ > 2σ(*F*^2^)] = 0.016	*w* = 1/[σ^2^(*F*_o_^2^) + (0.0128*P*)^2^] where *P* = (*F*_o_^2^ + 2*F*_c_^2^)/3
*wR*(*F*^2^) = 0.032	(Δ/σ)_max_ = 0.001
*S* = 1.01	Δρ_max_ = 0.40 e Å^−^^3^
3350 reflections	Δρ_min_ = −0.44 e Å^−^^3^
157 parameters	Extinction correction: SHELXL-2018/1 (Sheldrick 2015b), Fc^*^=kFc[1+0.001xFc^2^λ^3^/sin(2θ)]^-1/4^
0 restraints	Extinction coefficient: 0.0070 (6)
Primary atom site location: dual	

### Di-µ-chlorido-bis[heptaaqualanthanum(III)] tetrachloride (La) Special details

**Table:**

Geometry. All esds (except the esd in the dihedral angle between two l.s. planes) are estimated using the full covariance matrix. The cell esds are taken into account individually in the estimation of esds in distances, angles and torsion angles; correlations between esds in cell parameters are only used when they are defined by crystal symmetry. An approximate (isotropic) treatment of cell esds is used for estimating esds involving l.s. planes.

### Di-µ-chlorido-bis[heptaaqualanthanum(III)] tetrachloride (La) Fractional atomic coordinates and isotropic or equivalent isotropic displacement parameters (Å^2^)

**Table:**

	*x*	*y*	*z*	*U*_iso_*/*U*_eq_	
La1	0.21799 (2)	0.18615 (2)	0.32361 (2)	0.00928 (4)	
Cl1	−0.14698 (6)	0.17974 (5)	0.51434 (5)	0.01189 (8)	
Cl2	0.21093 (6)	0.77753 (5)	0.03491 (5)	0.01465 (8)	
Cl3	0.34270 (6)	0.38778 (5)	0.71161 (5)	0.01506 (9)	
O1	0.0304 (2)	0.42402 (17)	0.17472 (17)	0.0149 (3)	
O2	0.07092 (19)	0.08113 (17)	0.16194 (16)	0.0148 (3)	
O3	0.2234 (2)	0.46276 (18)	0.38494 (18)	0.0188 (3)	
O4	0.2311 (2)	0.09299 (18)	0.61748 (16)	0.0153 (3)	
O5	0.3868 (2)	0.35162 (17)	0.04773 (16)	0.0145 (3)	
O6	0.5212 (2)	0.2280 (2)	0.34138 (17)	0.0173 (3)	
O7	0.4532 (2)	−0.01065 (18)	0.19716 (17)	0.0164 (3)	
H1A	−0.060 (4)	0.447 (3)	0.219 (3)	0.030 (7)*	
H1B	0.077 (4)	0.509 (3)	0.119 (3)	0.028 (7)*	
H2A	0.015 (4)	0.143 (3)	0.103 (3)	0.033 (7)*	
H2B	0.116 (4)	0.003 (4)	0.131 (3)	0.035 (7)*	
H3A	0.259 (4)	0.470 (4)	0.458 (3)	0.042 (8)*	
H3B	0.199 (4)	0.553 (4)	0.330 (4)	0.051 (9)*	
H4A	0.144 (4)	0.052 (3)	0.677 (3)	0.027 (7)*	
H4B	0.261 (4)	0.171 (3)	0.644 (3)	0.030 (7)*	
H5A	0.360 (4)	0.367 (4)	−0.033 (3)	0.038 (8)*	
H5B	0.485 (4)	0.339 (4)	0.031 (4)	0.046 (9)*	
H6A	0.549 (5)	0.312 (4)	0.344 (4)	0.059 (11)*	
H6B	0.596 (4)	0.151 (4)	0.352 (3)	0.048 (9)*	
H7A	0.535 (4)	0.031 (4)	0.131 (3)	0.036 (8)*	
H7B	0.464 (5)	−0.115 (4)	0.228 (4)	0.057 (10)*	

### Di-µ-chlorido-bis[heptaaqualanthanum(III)] tetrachloride (La) Atomic displacement parameters (Å^2^)

**Table:**

	*U*^11^	*U*^22^	*U*^33^	*U*^12^	*U*^13^	*U*^23^
La1	0.00924 (5)	0.00899 (5)	0.00984 (5)	−0.00066 (3)	−0.00284 (3)	−0.00281 (3)
Cl1	0.0113 (2)	0.01008 (16)	0.01293 (17)	−0.00054 (14)	−0.00208 (15)	−0.00269 (14)
Cl2	0.0132 (2)	0.01316 (18)	0.01800 (19)	−0.00013 (15)	−0.00398 (16)	−0.00561 (15)
Cl3	0.0163 (2)	0.01438 (18)	0.01599 (19)	0.00004 (16)	−0.00586 (17)	−0.00548 (15)
O1	0.0113 (7)	0.0128 (6)	0.0165 (6)	−0.0003 (5)	−0.0020 (5)	−0.0007 (5)
O2	0.0168 (7)	0.0134 (6)	0.0173 (6)	0.0035 (5)	−0.0082 (6)	−0.0072 (5)
O3	0.0242 (8)	0.0134 (6)	0.0228 (7)	0.0012 (6)	−0.0101 (6)	−0.0083 (6)
O4	0.0155 (7)	0.0178 (6)	0.0135 (6)	−0.0060 (5)	−0.0035 (6)	−0.0041 (5)
O5	0.0128 (7)	0.0175 (6)	0.0122 (6)	−0.0009 (5)	−0.0036 (5)	−0.0028 (5)
O6	0.0130 (7)	0.0187 (7)	0.0231 (7)	−0.0009 (6)	−0.0062 (6)	−0.0086 (6)
O7	0.0157 (8)	0.0126 (6)	0.0187 (7)	−0.0007 (5)	−0.0016 (6)	−0.0044 (5)

### Di-µ-chlorido-bis[heptaaqualanthanum(III)] tetrachloride (La) Geometric parameters (Å, º)

**Table:**

La1—Cl1^i^	2.9376 (4)	O2—H2B	0.79 (3)
La1—Cl1	2.9187 (5)	O3—H3A	0.82 (3)
La1—O1	2.5443 (13)	O3—H3B	0.78 (3)
La1—O2	2.5504 (13)	O4—H4A	0.78 (3)
La1—O3	2.5315 (13)	O4—H4B	0.84 (3)
La1—O4	2.5873 (13)	O5—H5A	0.80 (3)
La1—O5	2.5233 (14)	O5—H5B	0.75 (3)
La1—O6	2.5413 (15)	O6—H6A	0.77 (3)
La1—O7	2.5643 (13)	O6—H6B	0.80 (3)
O1—H1A	0.75 (3)	O7—H7A	0.78 (3)
O1—H1B	0.79 (3)	O7—H7B	0.81 (3)
O2—H2A	0.80 (3)		
			
Cl1—La1—Cl1^i^	73.941 (12)	O6—La1—O1	123.86 (5)
O1—La1—Cl1^i^	128.72 (4)	O6—La1—O2	141.04 (4)
O1—La1—Cl1	69.96 (4)	O6—La1—O4	69.20 (5)
O1—La1—O2	67.52 (4)	O6—La1—O7	69.14 (5)
O1—La1—O4	130.45 (4)	O7—La1—Cl1^i^	68.54 (4)
O1—La1—O7	124.76 (4)	O7—La1—Cl1	139.55 (4)
O2—La1—Cl1	79.41 (3)	O7—La1—O4	104.74 (4)
O2—La1—Cl1^i^	71.07 (3)	La1—Cl1—La1^i^	106.059 (12)
O2—La1—O4	135.97 (5)	La1—O1—H1A	119 (2)
O2—La1—O7	74.71 (4)	La1—O1—H1B	117 (2)
O3—La1—Cl1^i^	140.68 (4)	H1A—O1—H1B	109 (3)
O3—La1—Cl1	84.97 (4)	La1—O2—H2A	124.0 (19)
O3—La1—O1	70.08 (5)	La1—O2—H2B	119 (2)
O3—La1—O2	137.58 (4)	H2A—O2—H2B	110 (2)
O3—La1—O4	74.18 (5)	La1—O3—H3A	125.3 (19)
O3—La1—O6	68.30 (5)	La1—O3—H3B	123 (2)
O3—La1—O7	134.49 (5)	H3A—O3—H3B	112 (3)
O4—La1—Cl1^i^	68.28 (3)	La1—O4—H4A	112.1 (19)
O4—La1—Cl1	73.83 (4)	La1—O4—H4B	114.3 (17)
O5—La1—Cl1	136.61 (3)	H4A—O4—H4B	112 (2)
O5—La1—Cl1^i^	133.83 (3)	La1—O5—H5A	126 (2)
O5—La1—O1	66.80 (5)	La1—O5—H5B	116 (2)
O5—La1—O2	81.23 (5)	H5A—O5—H5B	108 (3)
O5—La1—O3	83.78 (5)	La1—O6—H6A	124 (3)
O5—La1—O4	140.90 (5)	La1—O6—H6B	123 (2)
O5—La1—O6	72.77 (5)	H6A—O6—H6B	112 (3)
O5—La1—O7	68.91 (5)	La1—O7—H7A	119 (2)
O6—La1—Cl1	138.86 (3)	La1—O7—H7B	128 (2)
O6—La1—Cl1^i^	107.32 (4)	H7A—O7—H7B	113 (3)

Symmetry code: (i) −*x*, −*y*, −*z*+1.

### Di-µ-chlorido-bis[heptaaqualanthanum(III)] tetrachloride (La) Hydrogen-bond geometry (Å, º)

**Table:**

*D*—H···*A*	*D*—H	H···*A*	*D*···*A*	*D*—H···*A*
O1—H1*A*···Cl3^ii^	0.75 (3)	2.48 (3)	3.1947 (15)	161 (3)
O1—H1*B*···Cl2	0.79 (3)	2.38 (3)	3.1365 (16)	161 (2)
O2—H2*A*···Cl2^iii^	0.80 (3)	2.38 (3)	3.1378 (14)	157 (3)
O2—H2*B*···Cl2^iv^	0.79 (3)	2.26 (3)	3.0382 (13)	172 (3)
O3—H3*A*···Cl3	0.82 (3)	2.47 (3)	3.2552 (15)	160 (3)
O3—H3*B*···Cl1^ii^	0.78 (3)	2.92 (3)	3.2954 (13)	112 (2)
O3—H3*B*···Cl2	0.78 (3)	2.72 (3)	3.4160 (16)	150 (3)
O4—H4*A*···O2^i^	0.78 (3)	2.08 (3)	2.859 (2)	174 (3)
O4—H4*B*···Cl3	0.84 (3)	2.31 (3)	3.1488 (15)	179 (2)
O5—H5*A*···Cl3^v^	0.80 (3)	2.34 (3)	3.1308 (14)	167 (3)
O5—H5*B*···Cl2^vi^	0.75 (3)	2.44 (3)	3.1690 (15)	163 (3)
O5—H5*B*···O5^vi^	0.75 (3)	2.67 (3)	2.986 (3)	108 (2)
O6—H6*A*···Cl3^vii^	0.77 (3)	2.57 (3)	3.3288 (16)	168 (3)
O6—H6*B*···Cl1^viii^	0.80 (3)	2.93 (3)	3.3705 (14)	117 (2)
O6—H6*B*···O4^ix^	0.80 (3)	2.24 (3)	3.038 (2)	169 (3)

Symmetry codes: (i) −*x*, −*y*, −*z*+1; (ii) −*x*, −*y*+1, −*z*+1; (iii) −*x*, −*y*+1, −*z*; (iv) *x*, *y*−1, *z*; (v) *x*, *y*, *z*−1; (vi) −*x*+1, −*y*+1, −*z*; (vii) −*x*+1, −*y*+1, −*z*+1; (viii) *x*+1, *y*, *z*; (ix) −*x*+1, −*y*, −*z*+1.

### Di-µ-chlorido-bis[heptaaquacerium(III)] tetrachloride, (Ce) Crystal data

**Table:**

[CeCl(H_2_O)_7_]Cl_2_	*Z* = 2
*M**_r_* = 372.58	*F*(000) = 358
Triclinic, *P*1	*D*_x_ = 2.303 Mg m^−^^3^
*a* = 7.8995 (3) Å	Ag *K*α radiation, λ = 0.56086 Å
*b* = 8.2129 (3) Å	Cell parameters from 9812 reflections
*c* = 9.1961 (3) Å	θ = 2.5–23.7°
α = 70.451 (1)°	µ = 2.60 mm^−^^1^
β = 73.172 (1)°	*T* = 100 K
γ = 81.658 (1)°	Plate, colorless
*V* = 537.39 (3) Å^3^	0.26 × 0.22 × 0.17 mm

### Di-µ-chlorido-bis[heptaaquacerium(III)] tetrachloride, (Ce) Data collection

**Table:**

Bruker D8 Venture Duo diffractometer	3239 reflections with *I* > 2σ(*I*)
ω scans	*R*_int_ = 0.031
Absorption correction: multi-scan SADABS-2016/2 (Bruker, 2016)	θ_max_ = 23.7°, θ_min_ = 1.9°
*T*_min_ = 0.214, *T*_max_ = 0.257	*h* = −11→11
28063 measured reflections	*k* = −11→11
3304 independent reflections	*l* = −13→13

### Di-µ-chlorido-bis[heptaaquacerium(III)] tetrachloride, (Ce) Refinement

**Table:**

Refinement on *F*^2^	Hydrogen site location: difference Fourier map
Least-squares matrix: full	All H-atom parameters refined
*R*[*F*^2^ > 2σ(*F*^2^)] = 0.009	*w* = 1/[σ^2^(*F*_o_^2^) + (0.0036*P*)^2^ + 0.0733*P*] where *P* = (*F*_o_^2^ + 2*F*_c_^2^)/3
*wR*(*F*^2^) = 0.019	(Δ/σ)_max_ = 0.001
*S* = 1.17	Δρ_max_ = 0.32 e Å^−^^3^
3304 reflections	Δρ_min_ = −0.29 e Å^−^^3^
157 parameters	Extinction correction: SHELXL-2018/1 (Sheldrick 2015b), Fc^*^=kFc[1+0.001xFc^2^λ^3^/sin(2θ)]^-1/4^
0 restraints	Extinction coefficient: 0.0349 (8)
Primary atom site location: dual	

### Di-µ-chlorido-bis[heptaaquacerium(III)] tetrachloride, (Ce) Special details

**Table:**

Geometry. All esds (except the esd in the dihedral angle between two l.s. planes) are estimated using the full covariance matrix. The cell esds are taken into account individually in the estimation of esds in distances, angles and torsion angles; correlations between esds in cell parameters are only used when they are defined by crystal symmetry. An approximate (isotropic) treatment of cell esds is used for estimating esds involving l.s. planes.

### Di-µ-chlorido-bis[heptaaquacerium(III)] tetrachloride, (Ce) Fractional atomic coordinates and isotropic or equivalent isotropic displacement parameters (Å^2^)

**Table:**

	*x*	*y*	*z*	*U*_iso_*/*U*_eq_	
Ce1	0.21779 (2)	0.18632 (2)	0.32382 (2)	0.00590 (2)	
Cl1	−0.14612 (3)	0.17948 (3)	0.51435 (3)	0.00867 (4)	
Cl2	0.21050 (3)	0.77796 (3)	0.03514 (3)	0.01125 (4)	
Cl3	0.34287 (3)	0.38691 (3)	0.71200 (3)	0.01174 (4)	
O1	0.03034 (11)	0.42224 (10)	0.17488 (9)	0.01184 (13)	
O2	0.07253 (10)	0.08169 (10)	0.16291 (9)	0.01131 (13)	
O3	0.22060 (11)	0.46047 (10)	0.38631 (10)	0.01504 (14)	
O4	0.23110 (10)	0.09321 (10)	0.61518 (9)	0.01193 (13)	
O5	0.38549 (10)	0.35125 (10)	0.04929 (8)	0.01117 (13)	
O6	0.52006 (10)	0.22922 (11)	0.34141 (9)	0.01312 (14)	
O7	0.45125 (10)	−0.00898 (10)	0.19850 (9)	0.01262 (14)	
H1A	−0.062 (3)	0.449 (2)	0.218 (2)	0.034 (5)*	
H1B	0.074 (2)	0.507 (2)	0.120 (2)	0.034 (5)*	
H2A	0.122 (2)	0.003 (2)	0.132 (2)	0.034 (5)*	
H2B	0.020 (2)	0.144 (2)	0.103 (2)	0.033 (4)*	
H3A	0.251 (3)	0.468 (2)	0.458 (2)	0.042 (5)*	
H3B	0.191 (3)	0.554 (3)	0.333 (2)	0.050 (6)*	
H4A	0.142 (2)	0.055 (2)	0.6783 (19)	0.023 (4)*	
H4B	0.255 (2)	0.162 (2)	0.6465 (19)	0.028 (4)*	
H5A	0.353 (2)	0.362 (2)	−0.028 (2)	0.026 (4)*	
H5B	0.487 (3)	0.348 (2)	0.027 (2)	0.034 (5)*	
H6A	0.602 (3)	0.156 (2)	0.343 (2)	0.035 (5)*	
H6B	0.553 (3)	0.312 (3)	0.347 (2)	0.044 (5)*	
H7A	0.535 (2)	0.030 (2)	0.134 (2)	0.028 (4)*	
H7B	0.472 (2)	−0.108 (3)	0.232 (2)	0.039 (5)*	

### Di-µ-chlorido-bis[heptaaquacerium(III)] tetrachloride, (Ce) Atomic displacement parameters (Å^2^)

**Table:**

	*U*^11^	*U*^22^	*U*^33^	*U*^12^	*U*^13^	*U*^23^
Ce1	0.00610 (3)	0.00554 (3)	0.00634 (3)	−0.00042 (2)	−0.00203 (2)	−0.00178 (2)
Cl1	0.00832 (9)	0.00669 (9)	0.00983 (9)	−0.00012 (7)	−0.00148 (7)	−0.00192 (7)
Cl2	0.01010 (10)	0.00970 (10)	0.01461 (10)	0.00029 (8)	−0.00330 (8)	−0.00484 (8)
Cl3	0.01327 (10)	0.01078 (10)	0.01287 (10)	0.00063 (8)	−0.00533 (8)	−0.00471 (8)
O1	0.0101 (3)	0.0093 (3)	0.0125 (3)	0.0001 (3)	−0.0021 (3)	0.0003 (3)
O2	0.0137 (3)	0.0101 (3)	0.0124 (3)	0.0033 (3)	−0.0068 (3)	−0.0051 (3)
O3	0.0198 (4)	0.0105 (3)	0.0190 (4)	0.0010 (3)	−0.0093 (3)	−0.0070 (3)
O4	0.0126 (3)	0.0141 (3)	0.0105 (3)	−0.0051 (3)	−0.0029 (3)	−0.0040 (3)
O5	0.0093 (3)	0.0133 (3)	0.0096 (3)	−0.0008 (3)	−0.0022 (3)	−0.0019 (3)
O6	0.0090 (3)	0.0151 (3)	0.0178 (3)	−0.0006 (3)	−0.0049 (3)	−0.0071 (3)
O7	0.0103 (3)	0.0086 (3)	0.0157 (3)	0.0003 (3)	0.0003 (3)	−0.0031 (3)

### Di-µ-chlorido-bis[heptaaquacerium(III)] tetrachloride, (Ce) Geometric parameters (Å, º)

**Table:**

Ce1—Cl1^i^	2.9282 (2)	O2—H2B	0.783 (19)
Ce1—Cl1	2.8986 (2)	O3—H3A	0.79 (2)
Ce1—O1	2.5276 (7)	O3—H3B	0.80 (2)
Ce1—O2	2.5229 (7)	O4—H4A	0.800 (17)
Ce1—O3	2.5076 (8)	O4—H4B	0.781 (18)
Ce1—O4	2.5580 (7)	O5—H5A	0.798 (17)
Ce1—O5	2.5018 (7)	O5—H5B	0.768 (19)
Ce1—O6	2.5214 (7)	O6—H6A	0.82 (2)
Ce1—O7	2.5397 (8)	O6—H6B	0.79 (2)
O1—H1A	0.762 (19)	O7—H7A	0.775 (18)
O1—H1B	0.77 (2)	O7—H7B	0.78 (2)
O2—H2A	0.797 (19)		
			
Cl1—Ce1—Cl1^i^	73.953 (7)	O6—Ce1—O1	123.87 (3)
O1—Ce1—Cl1^i^	128.491 (19)	O6—Ce1—O2	140.89 (3)
O1—Ce1—Cl1	70.026 (19)	O6—Ce1—O4	69.23 (2)
O1—Ce1—O4	130.70 (2)	O6—Ce1—O7	69.36 (3)
O1—Ce1—O7	124.61 (3)	O7—Ce1—Cl1^i^	68.459 (19)
O2—Ce1—Cl1	79.796 (18)	O7—Ce1—Cl1	139.540 (18)
O2—Ce1—Cl1^i^	70.981 (18)	O7—Ce1—O4	104.66 (3)
O2—Ce1—O1	67.44 (2)	Ce1—Cl1—Ce1^i^	106.047 (7)
O2—Ce1—O4	135.98 (2)	Ce1—O1—H1A	120.9 (13)
O2—Ce1—O7	74.40 (3)	Ce1—O1—H1B	117.9 (13)
O3—Ce1—Cl1^i^	140.40 (2)	H1A—O1—H1B	105.8 (18)
O3—Ce1—Cl1	84.317 (19)	Ce1—O2—H2A	117.0 (13)
O3—Ce1—O1	70.16 (3)	Ce1—O2—H2B	123.0 (12)
O3—Ce1—O2	137.56 (3)	H2A—O2—H2B	111.1 (17)
O3—Ce1—O4	74.09 (3)	Ce1—O3—H3A	125.9 (14)
O3—Ce1—O6	68.59 (3)	Ce1—O3—H3B	122.9 (14)
O3—Ce1—O7	135.12 (3)	H3A—O3—H3B	111.1 (19)
O4—Ce1—Cl1^i^	68.263 (18)	Ce1—O4—H4A	114.1 (11)
O4—Ce1—Cl1	73.723 (18)	Ce1—O4—H4B	117.9 (12)
O5—Ce1—Cl1	136.599 (19)	H4A—O4—H4B	104.8 (15)
O5—Ce1—Cl1^i^	133.776 (18)	Ce1—O5—H5A	122.1 (12)
O5—Ce1—O1	66.70 (3)	Ce1—O5—H5B	120.1 (13)
O5—Ce1—O2	81.00 (2)	H5A—O5—H5B	108.9 (17)
O5—Ce1—O3	84.25 (3)	Ce1—O6—H6A	124.5 (12)
O5—Ce1—O4	141.04 (3)	Ce1—O6—H6B	127.8 (15)
O5—Ce1—O6	72.84 (2)	H6A—O6—H6B	107.7 (18)
O5—Ce1—O7	69.00 (3)	Ce1—O7—H7A	119.7 (12)
O6—Ce1—Cl1	138.654 (18)	Ce1—O7—H7B	129.6 (14)
O6—Ce1—Cl1^i^	107.53 (2)	H7A—O7—H7B	107.2 (18)

Symmetry code: (i) −*x*, −*y*, −*z*+1.

### Di-µ-chlorido-bis[heptaaquacerium(III)] tetrachloride, (Ce) Hydrogen-bond geometry (Å, º)

**Table:**

*D*—H···*A*	*D*—H	H···*A*	*D*···*A*	*D*—H···*A*
O1—H1*A*···Cl3^ii^	0.762 (19)	2.458 (19)	3.1945 (8)	162.9 (18)
O1—H1*B*···Cl2	0.77 (2)	2.405 (19)	3.1380 (8)	160.6 (17)
O2—H2*A*···Cl2^iii^	0.797 (19)	2.251 (19)	3.0368 (8)	168.6 (17)
O2—H2*B*···Cl2^iv^	0.783 (19)	2.411 (19)	3.1383 (8)	155.0 (16)
O3—H3*A*···Cl3	0.79 (2)	2.50 (2)	3.2501 (9)	160.9 (18)
O3—H3*B*···Cl1^ii^	0.80 (2)	2.91 (2)	3.3040 (8)	112.9 (16)
O3—H3*B*···Cl2	0.80 (2)	2.71 (2)	3.4188 (9)	147.3 (18)
O4—H4*A*···O2^i^	0.800 (17)	2.068 (17)	2.8609 (11)	171.2 (16)
O4—H4*B*···Cl3	0.781 (18)	2.369 (18)	3.1466 (8)	173.7 (16)
O5—H5*A*···Cl3^v^	0.798 (17)	2.354 (17)	3.1286 (8)	164.0 (16)
O5—H5*B*···Cl2^vi^	0.768 (19)	2.446 (19)	3.1699 (8)	157.6 (17)
O5—H5*B*···O5^vi^	0.768 (19)	2.601 (17)	2.9858 (15)	112.9 (15)
O6—H6*A*···Cl1^vii^	0.82 (2)	2.936 (18)	3.3662 (8)	115.2 (14)
O6—H6*A*···O4^viii^	0.82 (2)	2.25 (2)	3.0451 (11)	164.9 (17)
O6—H6*B*···Cl3^ix^	0.79 (2)	2.55 (2)	3.3165 (8)	164.4 (18)

Symmetry codes: (i) −*x*, −*y*, −*z*+1; (ii) −*x*, −*y*+1, −*z*+1; (iii) *x*, *y*−1, *z*; (iv) −*x*, −*y*+1, −*z*; (v) *x*, *y*, *z*−1; (vi) −*x*+1, −*y*+1, −*z*; (vii) *x*+1, *y*, *z*; (viii) −*x*+1, −*y*, −*z*+1; (ix) −*x*+1, −*y*+1, −*z*+1.

### Hexaaquadichloridoprotactinium(III) chloride (Pr) Crystal data

**Table:**

[PrCl_2_(H_2_O)_6_]Cl	*F*(000) = 340
*M**_r_* = 355.36	*D*_x_ = 2.323 Mg m^−^^3^
Monoclinic, *P*2/*c*	Ag *K*α radiation, λ = 0.56086 Å
*a* = 7.9997 (3) Å	Cell parameters from 6065 reflections
*b* = 6.5647 (2) Å	θ = 2.5–23.7°
*c* = 12.1734 (4) Å	µ = 2.90 mm^−^^1^
β = 127.388 (1)°	*T* = 100 K
*V* = 507.95 (3) Å^3^	Plate, green
*Z* = 2	0.06 × 0.06 × 0.05 mm

### Hexaaquadichloridoprotactinium(III) chloride (Pr) Data collection

**Table:**

Bruker D8 Venture Duo diffractometer	1486 reflections with *I* > 2σ(*I*)
ω scans	*R*_int_ = 0.058
Absorption correction: multi-scan SADABS-2016/2 (Bruker, 2016)	θ_max_ = 23.7°, θ_min_ = 2.5°
*T*_min_ = 0.218, *T*_max_ = 0.257	*h* = −11→11
15235 measured reflections	*k* = −9→9
1567 independent reflections	*l* = −17→17

### Hexaaquadichloridoprotactinium(III) chloride (Pr) Refinement

**Table:**

Refinement on *F*^2^	Primary atom site location: dual
Least-squares matrix: full	Hydrogen site location: difference Fourier map
*R*[*F*^2^ > 2σ(*F*^2^)] = 0.014	All H-atom parameters refined
*wR*(*F*^2^) = 0.027	*w* = 1/[σ^2^(*F*_o_^2^) + (0.0063*P*)^2^] where *P* = (*F*_o_^2^ + 2*F*_c_^2^)/3
*S* = 1.05	(Δ/σ)_max_ < 0.001
1567 reflections	Δρ_max_ = 0.38 e Å^−^^3^
71 parameters	Δρ_min_ = −0.39 e Å^−^^3^
0 restraints	

### Hexaaquadichloridoprotactinium(III) chloride (Pr) Special details

**Table:**

Geometry. All esds (except the esd in the dihedral angle between two l.s. planes) are estimated using the full covariance matrix. The cell esds are taken into account individually in the estimation of esds in distances, angles and torsion angles; correlations between esds in cell parameters are only used when they are defined by crystal symmetry. An approximate (isotropic) treatment of cell esds is used for estimating esds involving l.s. planes.

### Hexaaquadichloridoprotactinium(III) chloride (Pr) Fractional atomic coordinates and isotropic or equivalent isotropic displacement parameters (Å^2^)

**Table:**

	*x*	*y*	*z*	*U*_iso_*/*U*_eq_	
Pr1	0.500000	0.14679 (2)	0.250000	0.00584 (3)	
Cl1	0.29528 (6)	−0.17308 (5)	0.05469 (4)	0.01042 (7)	
Cl2	0.000000	0.62270 (8)	0.250000	0.01100 (10)	
O1	0.1632 (2)	0.29975 (19)	0.05765 (13)	0.0114 (2)	
O2	0.2337 (2)	0.0446 (2)	0.28224 (14)	0.0120 (2)	
O3	0.4395 (2)	0.42625 (18)	0.35562 (13)	0.0116 (2)	
H1A	0.063 (4)	0.265 (4)	0.044 (3)	0.030 (7)*	
H1B	0.132 (4)	0.327 (3)	−0.015 (3)	0.027 (7)*	
H2A	0.251 (4)	0.083 (4)	0.348 (3)	0.032 (7)*	
H2B	0.192 (4)	−0.065 (4)	0.268 (2)	0.025 (6)*	
H3A	0.508 (4)	0.524 (4)	0.384 (2)	0.030 (7)*	
H3B	0.323 (4)	0.454 (4)	0.328 (3)	0.027 (7)*	

### Hexaaquadichloridoprotactinium(III) chloride (Pr) Atomic displacement parameters (Å^2^)

**Table:**

	*U*^11^	*U*^22^	*U*^33^	*U*^12^	*U*^13^	*U*^23^
Pr1	0.00553 (6)	0.00602 (5)	0.00598 (6)	0.000	0.00349 (5)	0.000
Cl1	0.00986 (19)	0.00979 (16)	0.01028 (17)	−0.00112 (13)	0.00542 (15)	−0.00229 (13)
Cl2	0.0102 (3)	0.0119 (2)	0.0114 (2)	0.000	0.0068 (2)	0.000
O1	0.0075 (6)	0.0150 (6)	0.0101 (6)	0.0006 (5)	0.0046 (5)	0.0029 (4)
O2	0.0139 (7)	0.0124 (6)	0.0140 (6)	−0.0029 (5)	0.0107 (6)	−0.0020 (5)
O3	0.0089 (6)	0.0101 (5)	0.0159 (6)	−0.0013 (5)	0.0076 (5)	−0.0040 (5)

### Hexaaquadichloridoprotactinium(III) chloride (Pr) Geometric parameters (Å, º)

**Table:**

Pr1—Cl1^i^	2.8312 (4)	Pr1—O3^i^	2.4513 (12)
Pr1—Cl1	2.8312 (4)	O1—H1A	0.75 (3)
Pr1—O1^i^	2.4677 (13)	O1—H1B	0.78 (3)
Pr1—O1	2.4677 (13)	O2—H2A	0.76 (3)
Pr1—O2	2.4818 (13)	O2—H2B	0.77 (2)
Pr1—O2^i^	2.4817 (13)	O3—H3A	0.78 (3)
Pr1—O3	2.4513 (12)	O3—H3B	0.80 (3)
			
Cl1—Pr1—Cl1^i^	84.250 (16)	O3^i^—Pr1—O1	69.35 (5)
O1—Pr1—Cl1^i^	147.16 (3)	O3—Pr1—O1	75.14 (4)
O1^i^—Pr1—Cl1	147.16 (3)	O3^i^—Pr1—O1^i^	75.14 (4)
O1^i^—Pr1—Cl1^i^	76.31 (3)	O3—Pr1—O1^i^	69.35 (5)
O1—Pr1—Cl1	76.31 (3)	O3—Pr1—O2^i^	138.35 (4)
O1^i^—Pr1—O1	131.98 (6)	O3^i^—Pr1—O2	138.35 (4)
O1—Pr1—O2^i^	120.50 (4)	O3^i^—Pr1—O2^i^	69.97 (4)
O1—Pr1—O2	73.29 (4)	O3—Pr1—O2	69.97 (4)
O1^i^—Pr1—O2^i^	73.29 (4)	O3^i^—Pr1—O3	83.09 (6)
O1^i^—Pr1—O2	120.50 (4)	Pr1—O1—H1A	119 (2)
O2^i^—Pr1—Cl1	77.35 (3)	Pr1—O1—H1B	125.5 (19)
O2—Pr1—Cl1^i^	77.35 (3)	H1A—O1—H1B	105 (3)
O2^i^—Pr1—Cl1^i^	79.51 (3)	Pr1—O2—H2A	117 (2)
O2—Pr1—Cl1	79.51 (3)	Pr1—O2—H2B	121.3 (18)
O2^i^—Pr1—O2	148.63 (6)	H2A—O2—H2B	108 (2)
O3—Pr1—Cl1	142.92 (3)	Pr1—O3—H3A	122.8 (18)
O3—Pr1—Cl1^i^	108.20 (3)	Pr1—O3—H3B	120.2 (17)
O3^i^—Pr1—Cl1	108.20 (3)	H3A—O3—H3B	109 (2)
O3^i^—Pr1—Cl1^i^	142.92 (3)		

Symmetry code: (i) −*x*+1, *y*, −*z*+1/2.

### Hexaaquadichloridoprotactinium(III) chloride (Pr) Hydrogen-bond geometry (Å, º)

**Table:**

*D*—H···*A*	*D*—H	H···*A*	*D*···*A*	*D*—H···*A*
O1—H1*A*···Cl1^ii^	0.75 (3)	2.42 (3)	3.1498 (14)	166 (3)
O1—H1*B*···Cl2^iii^	0.78 (3)	2.40 (3)	3.1706 (13)	172 (2)
O2—H2*A*···Cl1^iv^	0.76 (3)	2.40 (3)	3.1575 (13)	175 (2)
O2—H2*B*···Cl2^v^	0.77 (2)	2.49 (2)	3.2291 (14)	163 (2)
O3—H3*A*···Cl1^vi^	0.78 (3)	2.36 (3)	3.1303 (14)	174 (2)
O3—H3*B*···Cl2	0.80 (3)	2.41 (3)	3.1919 (14)	166 (2)

Symmetry codes: (ii) −*x*, −*y*, −*z*; (iii) −*x*, −*y*+1, −*z*; (iv) *x*, −*y*, *z*+1/2; (v) *x*, *y*−1, *z*; (vi) −*x*+1, *y*+1, −*z*+1/2.

### Hexaaquadichloridoneodymium(III) chloride (Nd) Crystal data

**Table:**

[NdCl_2_(H_2_O)_6_]Cl	*F*(000) = 342
*M**_r_* = 358.69	*D*_x_ = 2.368 Mg m^−^^3^
Monoclinic, *P*2/*c*	Ag *K*α radiation, λ = 0.56086 Å
*a* = 7.9710 (3) Å	Cell parameters from 9892 reflections
*b* = 6.5460 (2) Å	θ = 2.5–23.7°
*c* = 12.1246 (5) Å	µ = 3.11 mm^−^^1^
β = 127.324 (1)°	*T* = 100 K
*V* = 503.09 (3) Å^3^	Plate, purple
*Z* = 2	0.16 × 0.12 × 0.11 mm

### Hexaaquadichloridoneodymium(III) chloride (Nd) Data collection

**Table:**

Bruker D8 Venture Duo diffractometer	1536 reflections with *I* > 2σ(*I*)
ω scans	*R*_int_ = 0.036
Absorption correction: multi-scan SADABS-2016/2 (Bruker, 2016)	θ_max_ = 23.7°, θ_min_ = 2.5°
*T*_min_ = 0.201, *T*_max_ = 0.257	*h* = −11→11
23233 measured reflections	*k* = −9→9
1559 independent reflections	*l* = −17→17

### Hexaaquadichloridoneodymium(III) chloride (Nd) Refinement

**Table:**

Refinement on *F*^2^	Hydrogen site location: difference Fourier map
Least-squares matrix: full	All H-atom parameters refined
*R*[*F*^2^ > 2σ(*F*^2^)] = 0.007	*w* = 1/[σ^2^(*F*_o_^2^) + (0.0058*P*)^2^ + 0.0784*P*] where *P* = (*F*_o_^2^ + 2*F*_c_^2^)/3
*wR*(*F*^2^) = 0.017	(Δ/σ)_max_ = 0.001
*S* = 1.11	Δρ_max_ = 0.25 e Å^−^^3^
1559 reflections	Δρ_min_ = −0.30 e Å^−^^3^
72 parameters	Extinction correction: SHELXL-2018/1 (Sheldrick 2015b), Fc^*^=kFc[1+0.001xFc^2^λ^3^/sin(2θ)]^-1/4^
0 restraints	Extinction coefficient: 0.0145 (7)
Primary atom site location: dual	

### Hexaaquadichloridoneodymium(III) chloride (Nd) Special details

**Table:**

Geometry. All esds (except the esd in the dihedral angle between two l.s. planes) are estimated using the full covariance matrix. The cell esds are taken into account individually in the estimation of esds in distances, angles and torsion angles; correlations between esds in cell parameters are only used when they are defined by crystal symmetry. An approximate (isotropic) treatment of cell esds is used for estimating esds involving l.s. planes.

### Hexaaquadichloridoneodymium(III) chloride (Nd) Fractional atomic coordinates and isotropic or equivalent isotropic displacement parameters (Å^2^)

**Table:**

	*x*	*y*	*z*	*U*_iso_*/*U*_eq_	
Nd1	0.500000	0.14781 (2)	0.250000	0.00585 (3)	
Cl1	0.29595 (3)	−0.17132 (3)	0.05545 (2)	0.01031 (4)	
Cl2	0.000000	0.62320 (5)	0.250000	0.01090 (5)	
O1	0.16396 (10)	0.30014 (11)	0.05851 (7)	0.01173 (12)	
O2	0.23447 (10)	0.04554 (11)	0.28178 (8)	0.01176 (12)	
O3	0.44019 (11)	0.42581 (11)	0.35561 (7)	0.01172 (12)	
H1A	0.063 (2)	0.268 (3)	0.0458 (17)	0.031 (4)*	
H1B	0.137 (2)	0.327 (2)	−0.0083 (18)	0.024 (4)*	
H2A	0.246 (3)	0.083 (3)	0.3463 (18)	0.034 (4)*	
H2B	0.187 (2)	−0.061 (3)	0.2652 (16)	0.028 (4)*	
H3A	0.511 (2)	0.526 (3)	0.3820 (16)	0.029 (4)*	
H3B	0.327 (3)	0.463 (2)	0.3272 (16)	0.028 (4)*	

### Hexaaquadichloridoneodymium(III) chloride (Nd) Atomic displacement parameters (Å^2^)

**Table:**

	*U*^11^	*U*^22^	*U*^33^	*U*^12^	*U*^13^	*U*^23^
Nd1	0.00556 (3)	0.00614 (4)	0.00597 (4)	0.000	0.00356 (3)	0.000
Cl1	0.01013 (8)	0.00972 (9)	0.00982 (9)	−0.00096 (6)	0.00539 (7)	−0.00215 (7)
Cl2	0.01039 (12)	0.01175 (13)	0.01133 (13)	0.000	0.00699 (10)	0.000
O1	0.0084 (3)	0.0150 (3)	0.0102 (3)	0.0005 (2)	0.0048 (2)	0.0031 (2)
O2	0.0136 (3)	0.0115 (3)	0.0142 (3)	−0.0033 (2)	0.0105 (3)	−0.0026 (3)
O3	0.0102 (3)	0.0104 (3)	0.0154 (3)	−0.0013 (2)	0.0081 (2)	−0.0037 (2)

### Hexaaquadichloridoneodymium(III) chloride (Nd) Geometric parameters (Å, º)

**Table:**

Nd1—Cl1	2.8145 (2)	Nd1—O3^i^	2.4328 (7)
Nd1—Cl1^i^	2.8145 (2)	O1—H1A	0.749 (16)
Nd1—O1^i^	2.4532 (7)	O1—H1B	0.722 (17)
Nd1—O1	2.4532 (7)	O2—H2A	0.770 (18)
Nd1—O2^i^	2.4629 (6)	O2—H2B	0.760 (17)
Nd1—O2	2.4629 (6)	O3—H3A	0.793 (16)
Nd1—O3	2.4328 (7)	O3—H3B	0.782 (16)
			
Cl1^i^—Nd1—Cl1	84.154 (9)	O3^i^—Nd1—O1	69.38 (2)
O1—Nd1—Cl1	76.348 (18)	O3—Nd1—O1	75.17 (2)
O1^i^—Nd1—Cl1^i^	76.348 (18)	O3^i^—Nd1—O1^i^	75.17 (2)
O1^i^—Nd1—Cl1	147.078 (18)	O3—Nd1—O1^i^	69.38 (2)
O1—Nd1—Cl1^i^	147.077 (18)	O3—Nd1—O2	70.11 (2)
O1^i^—Nd1—O1	132.04 (3)	O3^i^—Nd1—O2^i^	70.11 (2)
O1—Nd1—O2	73.13 (2)	O3^i^—Nd1—O2	138.33 (2)
O1—Nd1—O2^i^	120.74 (2)	O3—Nd1—O2^i^	138.33 (2)
O1^i^—Nd1—O2	120.74 (2)	O3^i^—Nd1—O3	83.16 (3)
O1^i^—Nd1—O2^i^	73.13 (2)	Nd1—O1—H1A	119.8 (13)
O2—Nd1—Cl1^i^	77.397 (17)	Nd1—O1—H1B	125.2 (12)
O2^i^—Nd1—Cl1	77.398 (17)	H1A—O1—H1B	106.3 (16)
O2—Nd1—Cl1	79.318 (18)	Nd1—O2—H2A	119.3 (12)
O2^i^—Nd1—Cl1^i^	79.318 (18)	Nd1—O2—H2B	123.0 (12)
O2—Nd1—O2^i^	148.45 (3)	H2A—O2—H2B	107.0 (16)
O3—Nd1—Cl1^i^	108.189 (17)	Nd1—O3—H3A	120.5 (11)
O3—Nd1—Cl1	142.963 (17)	Nd1—O3—H3B	122.7 (11)
O3^i^—Nd1—Cl1^i^	142.964 (17)	H3A—O3—H3B	105.9 (15)
O3^i^—Nd1—Cl1	108.190 (17)		

Symmetry code: (i) −*x*+1, *y*, −*z*+1/2.

### Hexaaquadichloridoneodymium(III) chloride (Nd) Hydrogen-bond geometry (Å, º)

**Table:**

*D*—H···*A*	*D*—H	H···*A*	*D*···*A*	*D*—H···*A*
O1—H1*A*···Cl1^ii^	0.749 (16)	2.418 (16)	3.1491 (7)	165.6 (16)
O1—H1*B*···Cl2^iii^	0.722 (17)	2.451 (18)	3.1661 (8)	170.8 (15)
O2—H2*A*···Cl1^iv^	0.770 (18)	2.387 (18)	3.1552 (8)	175.3 (17)
O2—H2*B*···Cl2^v^	0.760 (17)	2.490 (17)	3.2267 (8)	164.0 (15)
O3—H3*A*···Cl1^vi^	0.793 (16)	2.337 (17)	3.1290 (7)	176.1 (15)
O3—H3*B*···Cl2	0.782 (16)	2.413 (16)	3.1894 (7)	172.0 (15)

Symmetry codes: (ii) −*x*, −*y*, −*z*; (iii) −*x*, −*y*+1, −*z*; (iv) *x*, −*y*, *z*+1/2; (v) *x*, *y*−1, *z*; (vi) −*x*+1, *y*+1, −*z*+1/2.

### Hexaaquadichloridosamarium(III) chloride (Sm) Crystal data

**Table:**

[SmCl_2_(H_2_O)_6_]Cl	*F*(000) = 346
*M**_r_* = 364.80	*D*_x_ = 2.430 Mg m^−^^3^
Monoclinic, *P*2/*c*	Ag *K*α radiation, λ = 0.56086 Å
*a* = 7.9375 (8) Å	Cell parameters from 9537 reflections
*b* = 6.5351 (7) Å	θ = 3.0–23.7°
*c* = 12.0713 (11) Å	µ = 3.51 mm^−^^1^
β = 127.217 (2)°	*T* = 100 K
*V* = 498.65 (9) Å^3^	Plate, yellow
*Z* = 2	0.25 × 0.21 × 0.18 mm

### Hexaaquadichloridosamarium(III) chloride (Sm) Data collection

**Table:**

Bruker D8 Venture Duo diffractometer	1533 reflections with *I* > 2σ(*I*)
ω scans	*R*_int_ = 0.046
Absorption correction: multi-scan SADABS-2016/2 (Bruker, 2016)	θ_max_ = 23.7°, θ_min_ = 2.5°
*T*_min_ = 0.204, *T*_max_ = 0.257	*h* = −11→11
27415 measured reflections	*k* = −9→9
1553 independent reflections	*l* = −17→16

### Hexaaquadichloridosamarium(III) chloride (Sm) Refinement

**Table:**

Refinement on *F*^2^	Hydrogen site location: difference Fourier map
Least-squares matrix: full	All H-atom parameters refined
*R*[*F*^2^ > 2σ(*F*^2^)] = 0.010	*w* = 1/[σ^2^(*F*_o_^2^) + (0.0104*P*)^2^] where *P* = (*F*_o_^2^ + 2*F*_c_^2^)/3
*wR*(*F*^2^) = 0.023	(Δ/σ)_max_ = 0.001
*S* = 1.19	Δρ_max_ = 0.48 e Å^−^^3^
1553 reflections	Δρ_min_ = −0.85 e Å^−^^3^
72 parameters	Extinction correction: SHELXL-2018/1 (Sheldrick 2015b), Fc^*^=kFc[1+0.001xFc^2^λ^3^/sin(2θ)]^-1/4^
0 restraints	Extinction coefficient: 0.0299 (11)
Primary atom site location: dual	

### Hexaaquadichloridosamarium(III) chloride (Sm) Special details

**Table:**

Geometry. All esds (except the esd in the dihedral angle between two l.s. planes) are estimated using the full covariance matrix. The cell esds are taken into account individually in the estimation of esds in distances, angles and torsion angles; correlations between esds in cell parameters are only used when they are defined by crystal symmetry. An approximate (isotropic) treatment of cell esds is used for estimating esds involving l.s. planes.

### Hexaaquadichloridosamarium(III) chloride (Sm) Fractional atomic coordinates and isotropic or equivalent isotropic displacement parameters (Å^2^)

**Table:**

	*x*	*y*	*z*	*U*_iso_*/*U*_eq_	
Sm1	0.500000	0.14983 (2)	0.250000	0.00823 (4)	
Cl1	0.29725 (4)	−0.16793 (4)	0.05721 (3)	0.01414 (5)	
Cl2	0.000000	0.62416 (6)	0.250000	0.01522 (7)	
O1	0.16623 (14)	0.30030 (14)	0.06034 (9)	0.01545 (15)	
O2	0.23649 (13)	0.04788 (14)	0.28158 (9)	0.01546 (15)	
O3	0.44104 (14)	0.42539 (13)	0.35504 (9)	0.01546 (15)	
H1A	0.072 (3)	0.272 (3)	0.048 (2)	0.030 (5)*	
H1B	0.135 (4)	0.333 (3)	−0.013 (3)	0.039 (6)*	
H2A	0.250 (3)	0.085 (3)	0.351 (2)	0.028 (4)*	
H2B	0.187 (3)	−0.062 (3)	0.2644 (18)	0.024 (4)*	
H3A	0.510 (3)	0.525 (3)	0.381 (2)	0.041 (5)*	
H3B	0.324 (3)	0.457 (3)	0.3251 (18)	0.029 (4)*	

### Hexaaquadichloridosamarium(III) chloride (Sm) Atomic displacement parameters (Å^2^)

**Table:**

	*U*^11^	*U*^22^	*U*^33^	*U*^12^	*U*^13^	*U*^23^
Sm1	0.00783 (5)	0.00820 (5)	0.00880 (4)	0.000	0.00510 (3)	0.000
Cl1	0.01386 (12)	0.01289 (12)	0.01390 (11)	−0.00140 (8)	0.00746 (10)	−0.00317 (8)
Cl2	0.01447 (16)	0.01628 (16)	0.01618 (16)	0.000	0.00993 (14)	0.000
O1	0.0105 (4)	0.0191 (4)	0.0140 (4)	0.0011 (3)	0.0059 (3)	0.0040 (3)
O2	0.0174 (4)	0.0152 (4)	0.0192 (4)	−0.0043 (3)	0.0139 (3)	−0.0029 (3)
O3	0.0141 (4)	0.0131 (4)	0.0204 (4)	−0.0018 (3)	0.0111 (3)	−0.0052 (3)

### Hexaaquadichloridosamarium(III) chloride (Sm) Geometric parameters (Å, º)

**Table:**

Sm1—Cl1	2.7906 (3)	Sm1—O3	2.4057 (8)
Sm1—Cl1^i^	2.7906 (3)	O1—H1A	0.693 (19)
Sm1—O1^i^	2.4269 (8)	O1—H1B	0.79 (3)
Sm1—O1	2.4269 (8)	O2—H2A	0.81 (2)
Sm1—O2	2.4349 (8)	O2—H2B	0.78 (2)
Sm1—O2^i^	2.4349 (8)	O3—H3A	0.78 (2)
Sm1—O3^i^	2.4057 (8)	O3—H3B	0.794 (19)
			
Cl1^i^—Sm1—Cl1	83.829 (14)	O3—Sm1—O1	75.20 (3)
O1—Sm1—Cl1	76.40 (2)	O3^i^—Sm1—O1	69.44 (3)
O1^i^—Sm1—Cl1^i^	76.40 (2)	O3—Sm1—O1^i^	69.44 (3)
O1^i^—Sm1—Cl1	146.94 (2)	O3^i^—Sm1—O1^i^	75.20 (3)
O1—Sm1—Cl1^i^	146.94 (2)	O3^i^—Sm1—O2^i^	70.30 (3)
O1^i^—Sm1—O1	132.20 (4)	O3—Sm1—O2	70.30 (3)
O1—Sm1—O2^i^	120.94 (3)	O3—Sm1—O2^i^	138.31 (3)
O1—Sm1—O2	73.00 (3)	O3^i^—Sm1—O2	138.31 (3)
O1^i^—Sm1—O2^i^	73.00 (3)	O3—Sm1—O3^i^	83.07 (4)
O1^i^—Sm1—O2	120.94 (3)	Sm1—O1—H1A	120.8 (17)
O2^i^—Sm1—Cl1^i^	79.06 (2)	Sm1—O1—H1B	126.5 (17)
O2—Sm1—Cl1	79.06 (2)	H1A—O1—H1B	105 (2)
O2^i^—Sm1—Cl1	77.44 (2)	Sm1—O2—H2A	119.3 (13)
O2—Sm1—Cl1^i^	77.44 (2)	Sm1—O2—H2B	123.1 (13)
O2^i^—Sm1—O2	148.24 (4)	H2A—O2—H2B	106.3 (18)
O3^i^—Sm1—Cl1^i^	143.00 (2)	Sm1—O3—H3A	121.5 (16)
O3^i^—Sm1—Cl1	108.38 (2)	Sm1—O3—H3B	120.0 (13)
O3—Sm1—Cl1^i^	108.38 (2)	H3A—O3—H3B	108 (2)
O3—Sm1—Cl1	143.00 (2)		

Symmetry code: (i) −*x*+1, *y*, −*z*+1/2.

### Hexaaquadichloridosamarium(III) chloride (Sm) Hydrogen-bond geometry (Å, º)

**Table:**

*D*—H···*A*	*D*—H	H···*A*	*D*···*A*	*D*—H···*A*
O1—H1*A*···Cl1^ii^	0.693 (19)	2.484 (19)	3.1594 (10)	165 (2)
O1—H1*B*···Cl2^iii^	0.79 (3)	2.39 (3)	3.1685 (9)	169.2 (19)
O2—H2*A*···Cl1^iv^	0.81 (2)	2.35 (2)	3.1586 (9)	175.9 (18)
O2—H2*B*···Cl2^v^	0.78 (2)	2.48 (2)	3.2358 (10)	163.6 (17)
O3—H3*A*···Cl1^vi^	0.78 (2)	2.36 (2)	3.1367 (10)	176 (2)
O3—H3*B*···Cl2	0.794 (19)	2.41 (2)	3.1908 (9)	167.7 (18)

Symmetry codes: (ii) −*x*, −*y*, −*z*; (iii) −*x*, −*y*+1, −*z*; (iv) *x*, −*y*, *z*+1/2; (v) *x*, *y*−1, *z*; (vi) −*x*+1, *y*+1, −*z*+1/2.

### Hexaaquadichloridoeuropium(III) chloride (Eu) Crystal data

**Table:**

[EuCl_2_(H_2_O)_6_]Cl	*F*(000) = 348
*M**_r_* = 366.41	*D*_x_ = 2.466 Mg m^−^^3^
Monoclinic, *P*2/*c*	Ag *K*α radiation, λ = 0.56086 Å
*a* = 7.9062 (2) Å	Cell parameters from 9987 reflections
*b* = 6.5092 (2) Å	θ = 2.5–23.7°
*c* = 12.0410 (4) Å	µ = 3.75 mm^−^^1^
β = 127.231 (1)°	*T* = 100 K
*V* = 493.38 (3) Å^3^	Plate, colorless
*Z* = 2	0.13 × 0.13 × 0.11 mm

### Hexaaquadichloridoeuropium(III) chloride (Eu) Data collection

**Table:**

Bruker D8 Venture Duo diffractometer	1504 reflections with *I* > 2σ(*I*)
ω scans	*R*_int_ = 0.038
Absorption correction: multi-scan SADABS-2016/2 (Bruker, 2016)	θ_max_ = 23.7°, θ_min_ = 2.5°
*T*_min_ = 0.218, *T*_max_ = 0.257	*h* = −11→11
21905 measured reflections	*k* = −9→9
1529 independent reflections	*l* = −17→17

### Hexaaquadichloridoeuropium(III) chloride (Eu) Refinement

**Table:**

Refinement on *F*^2^	Hydrogen site location: difference Fourier map
Least-squares matrix: full	All H-atom parameters refined
*R*[*F*^2^ > 2σ(*F*^2^)] = 0.007	*w* = 1/[σ^2^(*F*_o_^2^) + (0.0058*P*)^2^] where *P* = (*F*_o_^2^ + 2*F*_c_^2^)/3
*wR*(*F*^2^) = 0.016	(Δ/σ)_max_ = 0.001
*S* = 1.13	Δρ_max_ = 0.29 e Å^−^^3^
1529 reflections	Δρ_min_ = −0.32 e Å^−^^3^
72 parameters	Extinction correction: SHELXL-2018/1 (Sheldrick 2015b), Fc^*^=kFc[1+0.001xFc^2^λ^3^/sin(2θ)]^-1/4^
0 restraints	Extinction coefficient: 0.0155 (6)
Primary atom site location: dual	

### Hexaaquadichloridoeuropium(III) chloride (Eu) Special details

**Table:**

Geometry. All esds (except the esd in the dihedral angle between two l.s. planes) are estimated using the full covariance matrix. The cell esds are taken into account individually in the estimation of esds in distances, angles and torsion angles; correlations between esds in cell parameters are only used when they are defined by crystal symmetry. An approximate (isotropic) treatment of cell esds is used for estimating esds involving l.s. planes.

### Hexaaquadichloridoeuropium(III) chloride (Eu) Fractional atomic coordinates and isotropic or equivalent isotropic displacement parameters (Å^2^)

**Table:**

	*x*	*y*	*z*	*U*_iso_*/*U*_eq_	
Eu1	0.500000	0.15065 (2)	0.250000	0.00549 (3)	
Cl1	0.29777 (3)	−0.16684 (3)	0.05730 (2)	0.00967 (4)	
Cl2	0.000000	0.62348 (4)	0.250000	0.01034 (6)	
O1	0.16667 (11)	0.30060 (11)	0.06078 (8)	0.01088 (13)	
O2	0.23665 (11)	0.04821 (11)	0.28100 (8)	0.01108 (13)	
O3	0.44219 (11)	0.42430 (10)	0.35568 (8)	0.01082 (13)	
H1A	0.062 (2)	0.268 (2)	0.0449 (17)	0.029 (4)*	
H1B	0.134 (3)	0.332 (2)	−0.011 (2)	0.028 (4)*	
H2A	0.250 (2)	0.085 (2)	0.3479 (17)	0.024 (4)*	
H2B	0.193 (2)	−0.060 (2)	0.2653 (16)	0.026 (4)*	
H3A	0.513 (2)	0.523 (2)	0.3791 (16)	0.030 (4)*	
H3B	0.334 (2)	0.458 (2)	0.3268 (16)	0.025 (4)*	

### Hexaaquadichloridoeuropium(III) chloride (Eu) Atomic displacement parameters (Å^2^)

**Table:**

	*U*^11^	*U*^22^	*U*^33^	*U*^12^	*U*^13^	*U*^23^
Eu1	0.00511 (3)	0.00572 (3)	0.00569 (4)	0.000	0.00329 (3)	0.000
Cl1	0.00927 (9)	0.00919 (9)	0.00941 (10)	−0.00089 (7)	0.00505 (8)	−0.00199 (7)
Cl2	0.00946 (13)	0.01139 (13)	0.01076 (14)	0.000	0.00643 (12)	0.000
O1	0.0075 (3)	0.0138 (3)	0.0098 (3)	0.0005 (2)	0.0044 (3)	0.0026 (3)
O2	0.0130 (3)	0.0106 (3)	0.0135 (3)	−0.0026 (2)	0.0101 (3)	−0.0020 (3)
O3	0.0083 (3)	0.0095 (3)	0.0147 (4)	−0.0013 (2)	0.0071 (3)	−0.0039 (3)

### Hexaaquadichloridoeuropium(III) chloride (Eu) Geometric parameters (Å, º)

**Table:**

Eu1—Cl1	2.7788 (2)	Eu1—O3	2.3884 (7)
Eu1—Cl1^i^	2.7788 (2)	O1—H1A	0.760 (15)
Eu1—O1^i^	2.4131 (7)	O1—H1B	0.766 (19)
Eu1—O1	2.4131 (7)	O2—H2A	0.784 (16)
Eu1—O2	2.4206 (7)	O2—H2B	0.759 (16)
Eu1—O2^i^	2.4206 (7)	O3—H3A	0.783 (16)
Eu1—O3^i^	2.3884 (7)	O3—H3B	0.737 (15)
			
Cl1^i^—Eu1—Cl1	83.904 (10)	O3—Eu1—O1	75.50 (3)
O1—Eu1—Cl1	76.376 (18)	O3^i^—Eu1—O1	69.33 (2)
O1^i^—Eu1—Cl1^i^	76.376 (18)	O3—Eu1—O1^i^	69.33 (2)
O1^i^—Eu1—Cl1	146.839 (18)	O3^i^—Eu1—O1^i^	75.50 (3)
O1—Eu1—Cl1^i^	146.840 (18)	O3^i^—Eu1—O2^i^	70.34 (2)
O1^i^—Eu1—O1	132.29 (3)	O3—Eu1—O2	70.34 (2)
O1—Eu1—O2^i^	121.05 (3)	O3—Eu1—O2^i^	138.36 (2)
O1—Eu1—O2	72.97 (3)	O3^i^—Eu1—O2	138.36 (2)
O1^i^—Eu1—O2^i^	72.97 (3)	O3—Eu1—O3^i^	83.55 (3)
O1^i^—Eu1—O2	121.05 (3)	Eu1—O1—H1A	121.7 (12)
O2^i^—Eu1—Cl1^i^	79.036 (19)	Eu1—O1—H1B	127.1 (12)
O2—Eu1—Cl1	79.037 (19)	H1A—O1—H1B	102.6 (16)
O2^i^—Eu1—Cl1	77.317 (17)	Eu1—O2—H2A	118.7 (11)
O2—Eu1—Cl1^i^	77.317 (17)	Eu1—O2—H2B	121.7 (11)
O2^i^—Eu1—O2	148.02 (3)	H2A—O2—H2B	107.3 (15)
O3^i^—Eu1—Cl1^i^	143.185 (18)	Eu1—O3—H3A	118.5 (11)
O3^i^—Eu1—Cl1	107.979 (18)	Eu1—O3—H3B	120.7 (12)
O3—Eu1—Cl1^i^	107.980 (18)	H3A—O3—H3B	107.7 (15)
O3—Eu1—Cl1	143.185 (18)		

Symmetry code: (i) −*x*+1, *y*, −*z*+1/2.

### Hexaaquadichloridoeuropium(III) chloride (Eu) Hydrogen-bond geometry (Å, º)

**Table:**

*D*—H···*A*	*D*—H	H···*A*	*D*···*A*	*D*—H···*A*
O1—H1*A*···Cl1^ii^	0.760 (15)	2.407 (15)	3.1537 (7)	167.5 (16)
O1—H1*B*···Cl2^iii^	0.766 (19)	2.407 (19)	3.1649 (8)	170.6 (14)
O2—H2*A*···Cl1^iv^	0.784 (16)	2.373 (17)	3.1549 (8)	175.1 (14)
O2—H2*B*···Cl2^v^	0.759 (16)	2.500 (15)	3.2308 (7)	162.1 (14)
O3—H3*A*···Cl1^vi^	0.783 (16)	2.349 (16)	3.1307 (7)	175.7 (14)
O3—H3*B*···Cl2	0.737 (15)	2.455 (15)	3.1842 (7)	170.3 (15)

Symmetry codes: (ii) −*x*, −*y*, −*z*; (iii) −*x*, −*y*+1, −*z*; (iv) *x*, −*y*, *z*+1/2; (v) *x*, *y*−1, *z*; (vi) −*x*+1, *y*+1, −*z*+1/2.

### Hexaaquadichloridogadolinium(III) chloride (Gd) Crystal data

**Table:**

[GdCl_2_(H_2_O)_6_]Cl	*F*(000) = 350
*M**_r_* = 371.70	*D*_x_ = 2.518 Mg m^−^^3^
Monoclinic, *P*2/*c*	Ag *K*α radiation, λ = 0.56086 Å
*a* = 7.8835 (3) Å	Cell parameters from 9213 reflections
*b* = 6.4964 (3) Å	θ = 2.5–23.8°
*c* = 12.0176 (4) Å	µ = 3.99 mm^−^^1^
β = 127.186 (1)°	*T* = 100 K
*V* = 490.33 (3) Å^3^	Plate, colorless
*Z* = 2	0.33 × 0.25 × 0.20 mm

### Hexaaquadichloridogadolinium(III) chloride (Gd) Data collection

**Table:**

Bruker D8 Venture Duo diffractometer	1520 reflections with *I* > 2σ(*I*)
ω scans	*R*_int_ = 0.037
Absorption correction: multi-scan SADABS-2016/2 (Bruker, 2016)	θ_max_ = 23.8°, θ_min_ = 2.5°
*T*_min_ = 0.172, *T*_max_ = 0.257	*h* = −11→11
26021 measured reflections	*k* = −9→9
1532 independent reflections	*l* = −17→17

### Hexaaquadichloridogadolinium(III) chloride (Gd) Refinement

**Table:**

Refinement on *F*^2^	Hydrogen site location: difference Fourier map
Least-squares matrix: full	All H-atom parameters refined
*R*[*F*^2^ > 2σ(*F*^2^)] = 0.009	*w* = 1/[σ^2^(*F*_o_^2^) + (0.0082*P*)^2^ + 0.0734*P*] where *P* = (*F*_o_^2^ + 2*F*_c_^2^)/3
*wR*(*F*^2^) = 0.021	(Δ/σ)_max_ = 0.001
*S* = 1.23	Δρ_max_ = 0.44 e Å^−^^3^
1532 reflections	Δρ_min_ = −0.38 e Å^−^^3^
72 parameters	Extinction correction: SHELXL-2018/1 (Sheldrick 2015b), Fc^*^=kFc[1+0.001xFc^2^λ^3^/sin(2θ)]^-1/4^
0 restraints	Extinction coefficient: 0.0279 (9)
Primary atom site location: dual	

### Hexaaquadichloridogadolinium(III) chloride (Gd) Special details

**Table:**

Geometry. All esds (except the esd in the dihedral angle between two l.s. planes) are estimated using the full covariance matrix. The cell esds are taken into account individually in the estimation of esds in distances, angles and torsion angles; correlations between esds in cell parameters are only used when they are defined by crystal symmetry. An approximate (isotropic) treatment of cell esds is used for estimating esds involving l.s. planes.

### Hexaaquadichloridogadolinium(III) chloride (Gd) Fractional atomic coordinates and isotropic or equivalent isotropic displacement parameters (Å^2^)

**Table:**

	*x*	*y*	*z*	*U*_iso_*/*U*_eq_	
Gd1	0.500000	0.15160 (2)	0.250000	0.00581 (3)	
Cl1	0.29831 (4)	−0.16561 (4)	0.05775 (3)	0.01005 (5)	
Cl2	0.000000	0.62367 (6)	0.250000	0.01075 (7)	
O1	0.16722 (14)	0.30084 (14)	0.06138 (9)	0.01150 (15)	
O2	0.23723 (13)	0.04881 (14)	0.28098 (9)	0.01135 (15)	
O3	0.44242 (14)	0.42417 (13)	0.35554 (9)	0.01117 (15)	
H1A	0.060 (3)	0.266 (3)	0.047 (2)	0.028 (5)*	
H1B	0.139 (3)	0.329 (3)	−0.008 (2)	0.023 (5)*	
H2A	0.254 (3)	0.086 (3)	0.350 (2)	0.029 (5)*	
H2B	0.189 (3)	−0.068 (3)	0.264 (2)	0.030 (5)*	
H3A	0.513 (4)	0.532 (3)	0.381 (2)	0.037 (5)*	
H3B	0.325 (3)	0.458 (3)	0.326 (2)	0.027 (5)*	

### Hexaaquadichloridogadolinium(III) chloride (Gd) Atomic displacement parameters (Å^2^)

**Table:**

	*U*^11^	*U*^22^	*U*^33^	*U*^12^	*U*^13^	*U*^23^
Gd1	0.00572 (4)	0.00572 (4)	0.00615 (4)	0.000	0.00367 (3)	0.000
Cl1	0.00989 (11)	0.00916 (11)	0.00986 (11)	−0.00088 (8)	0.00532 (10)	−0.00202 (8)
Cl2	0.01014 (16)	0.01129 (16)	0.01144 (16)	0.000	0.00685 (14)	0.000
O1	0.0083 (4)	0.0145 (4)	0.0100 (4)	0.0004 (3)	0.0046 (3)	0.0025 (3)
O2	0.0127 (4)	0.0110 (4)	0.0138 (4)	−0.0024 (3)	0.0098 (3)	−0.0017 (3)
O3	0.0100 (4)	0.0088 (4)	0.0151 (4)	−0.0010 (3)	0.0078 (3)	−0.0032 (3)

### Hexaaquadichloridogadolinium(III) chloride (Gd) Geometric parameters (Å, º)

**Table:**

Gd1—Cl1^i^	2.7699 (3)	Gd1—O3^i^	2.3762 (8)
Gd1—Cl1	2.7699 (3)	O1—H1A	0.79 (2)
Gd1—O1	2.4026 (9)	O1—H1B	0.75 (2)
Gd1—O1^i^	2.4026 (9)	O2—H2A	0.80 (2)
Gd1—O2^i^	2.4101 (8)	O2—H2B	0.82 (2)
Gd1—O2	2.4101 (8)	O3—H3A	0.83 (2)
Gd1—O3	2.3762 (8)	O3—H3B	0.79 (2)
			
Cl1—Gd1—Cl1^i^	83.855 (12)	O3^i^—Gd1—O1^i^	75.58 (3)
O1^i^—Gd1—Cl1^i^	76.37 (2)	O3—Gd1—O1^i^	69.37 (3)
O1—Gd1—Cl1	76.37 (2)	O3^i^—Gd1—O1	69.37 (3)
O1—Gd1—Cl1^i^	146.74 (2)	O3—Gd1—O1	75.58 (3)
O1^i^—Gd1—Cl1	146.74 (2)	O3—Gd1—O2	70.41 (3)
O1—Gd1—O1^i^	132.40 (4)	O3^i^—Gd1—O2^i^	70.41 (3)
O1^i^—Gd1—O2	121.09 (3)	O3^i^—Gd1—O2	138.44 (3)
O1^i^—Gd1—O2^i^	72.98 (3)	O3—Gd1—O2^i^	138.44 (3)
O1—Gd1—O2	72.97 (3)	O3^i^—Gd1—O3	83.65 (4)
O1—Gd1—O2^i^	121.09 (3)	Gd1—O1—H1A	120.3 (15)
O2—Gd1—Cl1	78.99 (2)	Gd1—O1—H1B	125.0 (15)
O2^i^—Gd1—Cl1^i^	78.99 (2)	H1A—O1—H1B	106 (2)
O2—Gd1—Cl1^i^	77.21 (2)	Gd1—O2—H2A	117.6 (15)
O2^i^—Gd1—Cl1	77.21 (2)	Gd1—O2—H2B	121.9 (14)
O2—Gd1—O2^i^	147.83 (4)	H2A—O2—H2B	108 (2)
O3—Gd1—Cl1	143.24 (2)	Gd1—O3—H3A	121.6 (14)
O3—Gd1—Cl1^i^	107.92 (2)	Gd1—O3—H3B	121.0 (14)
O3^i^—Gd1—Cl1	107.92 (2)	H3A—O3—H3B	106 (2)
O3^i^—Gd1—Cl1^i^	143.24 (2)		

Symmetry code: (i) −*x*+1, *y*, −*z*+1/2.

### Hexaaquadichloridogadolinium(III) chloride (Gd) Hydrogen-bond geometry (Å, º)

**Table:**

*D*—H···*A*	*D*—H	H···*A*	*D*···*A*	*D*—H···*A*
O1—H1*A*···Cl1^ii^	0.79 (2)	2.39 (2)	3.1527 (9)	165 (2)
O1—H1*B*···Cl2^iii^	0.75 (2)	2.43 (2)	3.1651 (9)	170.1 (19)
O2—H2*A*···Cl1^iv^	0.80 (2)	2.36 (2)	3.1519 (9)	175 (2)
O2—H2*B*···Cl2^v^	0.82 (2)	2.44 (2)	3.2284 (9)	161.7 (18)
O3—H3*A*···Cl1^vi^	0.83 (2)	2.30 (2)	3.1290 (9)	178 (2)
O3—H3*B*···Cl2	0.79 (2)	2.40 (2)	3.1790 (9)	169.1 (19)

Symmetry codes: (ii) −*x*, −*y*, −*z*; (iii) −*x*, −*y*+1, −*z*; (iv) *x*, −*y*, *z*+1/2; (v) *x*, *y*−1, *z*; (vi) −*x*+1, *y*+1, −*z*+1/2.

### Hexaaquadichloridoterbium(III) chloride (Tb) Crystal data

**Table:**

[TbCl_2_(H_2_O)_6_]Cl	*F*(000) = 352
*M**_r_* = 373.37	*D*_x_ = 2.542 Mg m^−^^3^
Monoclinic, *P*2/*c*	Ag *K*α radiation, λ = 0.56086 Å
*a* = 7.8646 (3) Å	Cell parameters from 9445 reflections
*b* = 6.4903 (3) Å	θ = 2.5–23.7°
*c* = 11.9871 (5) Å	µ = 4.24 mm^−^^1^
β = 127.134 (1)°	*T* = 100 K
*V* = 487.79 (4) Å^3^	Plate, colorless
*Z* = 2	0.3 × 0.16 × 0.12 mm

### Hexaaquadichloridoterbium(III) chloride (Tb) Data collection

**Table:**

Bruker D8 Venture Duo diffractometer	1481 reflections with *I* > 2σ(*I*)
ω scans	*R*_int_ = 0.024
Absorption correction: multi-scan SADABS-2016/2 (Bruker, 2016)	θ_max_ = 23.7°, θ_min_ = 2.5°
*T*_min_ = 0.223, *T*_max_ = 0.257	*h* = −10→11
11117 measured reflections	*k* = −9→9
1503 independent reflections	*l* = −17→17

### Hexaaquadichloridoterbium(III) chloride (Tb) Refinement

**Table:**

Refinement on *F*^2^	Hydrogen site location: difference Fourier map
Least-squares matrix: full	All H-atom parameters refined
*R*[*F*^2^ > 2σ(*F*^2^)] = 0.009	*w* = 1/[σ^2^(*F*_o_^2^) + (0.008*P*)^2^ + 0.0701*P*] where *P* = (*F*_o_^2^ + 2*F*_c_^2^)/3
*wR*(*F*^2^) = 0.020	(Δ/σ)_max_ < 0.001
*S* = 1.10	Δρ_max_ = 0.36 e Å^−^^3^
1503 reflections	Δρ_min_ = −0.37 e Å^−^^3^
72 parameters	Extinction correction: SHELXL-2018/1 (Sheldrick 2015b), Fc^*^=kFc[1+0.001xFc^2^λ^3^/sin(2θ)]^-1/4^
0 restraints	Extinction coefficient: 0.0117 (6)
Primary atom site location: dual	

### Hexaaquadichloridoterbium(III) chloride (Tb) Special details

**Table:**

Geometry. All esds (except the esd in the dihedral angle between two l.s. planes) are estimated using the full covariance matrix. The cell esds are taken into account individually in the estimation of esds in distances, angles and torsion angles; correlations between esds in cell parameters are only used when they are defined by crystal symmetry. An approximate (isotropic) treatment of cell esds is used for estimating esds involving l.s. planes.

### Hexaaquadichloridoterbium(III) chloride (Tb) Fractional atomic coordinates and isotropic or equivalent isotropic displacement parameters (Å^2^)

**Table:**

	*x*	*y*	*z*	*U*_iso_*/*U*_eq_	
Tb1	0.500000	0.15305 (2)	0.250000	0.00536 (3)	
Cl1	0.29840 (4)	−0.16404 (4)	0.05842 (3)	0.00937 (5)	
Cl2	0.000000	0.62554 (6)	0.250000	0.01012 (7)	
O1	0.16832 (14)	0.30085 (14)	0.06248 (10)	0.01064 (16)	
O2	0.23848 (14)	0.05048 (15)	0.28092 (10)	0.01085 (16)	
O3	0.44279 (14)	0.42471 (14)	0.35533 (9)	0.01056 (16)	
H1A	0.065 (3)	0.265 (3)	0.049 (2)	0.026 (5)*	
H1B	0.139 (3)	0.325 (3)	−0.011 (2)	0.028 (5)*	
H2A	0.257 (3)	0.088 (3)	0.353 (2)	0.030 (5)*	
H2B	0.194 (3)	−0.058 (3)	0.262 (2)	0.026 (5)*	
H3A	0.519 (3)	0.525 (3)	0.383 (2)	0.028 (5)*	
H3B	0.327 (3)	0.453 (3)	0.326 (2)	0.033 (5)*	

### Hexaaquadichloridoterbium(III) chloride (Tb) Atomic displacement parameters (Å^2^)

**Table:**

	*U*^11^	*U*^22^	*U*^33^	*U*^12^	*U*^13^	*U*^23^
Tb1	0.00486 (4)	0.00550 (4)	0.00570 (4)	0.000	0.00318 (3)	0.000
Cl1	0.00902 (11)	0.00878 (12)	0.00908 (11)	−0.00078 (8)	0.00481 (10)	−0.00186 (9)
Cl2	0.00953 (16)	0.01080 (17)	0.01046 (17)	0.000	0.00626 (14)	0.000
O1	0.0076 (4)	0.0132 (4)	0.0094 (4)	0.0004 (3)	0.0043 (3)	0.0024 (3)
O2	0.0123 (4)	0.0102 (4)	0.0132 (4)	−0.0030 (3)	0.0094 (3)	−0.0027 (3)
O3	0.0089 (4)	0.0088 (4)	0.0145 (4)	−0.0011 (3)	0.0073 (3)	−0.0035 (3)

### Hexaaquadichloridoterbium(III) chloride (Tb) Geometric parameters (Å, º)

**Table:**

Tb1—Cl1^i^	2.7617 (3)	Tb1—O3	2.3646 (9)
Tb1—Cl1	2.7617 (3)	O1—H1A	0.77 (2)
Tb1—O1	2.3870 (9)	O1—H1B	0.77 (2)
Tb1—O1^i^	2.3870 (9)	O2—H2A	0.82 (2)
Tb1—O2^i^	2.3940 (9)	O2—H2B	0.76 (2)
Tb1—O2	2.3940 (9)	O3—H3A	0.81 (2)
Tb1—O3^i^	2.3646 (9)	O3—H3B	0.77 (2)
			
Cl1—Tb1—Cl1^i^	83.649 (12)	O3—Tb1—O1^i^	69.48 (3)
O1^i^—Tb1—Cl1^i^	76.35 (2)	O3^i^—Tb1—O1^i^	75.59 (3)
O1—Tb1—Cl1	76.35 (2)	O3—Tb1—O1	75.59 (3)
O1—Tb1—Cl1^i^	146.61 (2)	O3^i^—Tb1—O1	69.49 (3)
O1^i^—Tb1—Cl1	146.61 (2)	O3^i^—Tb1—O2	138.44 (3)
O1—Tb1—O1^i^	132.61 (4)	O3—Tb1—O2^i^	138.44 (3)
O1^i^—Tb1—O2	121.23 (3)	O3—Tb1—O2	70.52 (3)
O1^i^—Tb1—O2^i^	72.84 (3)	O3^i^—Tb1—O2^i^	70.52 (3)
O1—Tb1—O2	72.84 (3)	O3—Tb1—O3^i^	83.57 (5)
O1—Tb1—O2^i^	121.24 (3)	Tb1—O1—H1A	119.7 (16)
O2—Tb1—Cl1	78.81 (2)	Tb1—O1—H1B	124.1 (16)
O2^i^—Tb1—Cl1^i^	78.81 (2)	H1A—O1—H1B	106 (2)
O2—Tb1—Cl1^i^	77.27 (2)	Tb1—O2—H2A	117.3 (14)
O2^i^—Tb1—Cl1	77.27 (2)	Tb1—O2—H2B	119.8 (15)
O2—Tb1—O2^i^	147.71 (5)	H2A—O2—H2B	110 (2)
O3^i^—Tb1—Cl1	108.08 (2)	Tb1—O3—H3A	119.5 (14)
O3^i^—Tb1—Cl1^i^	143.22 (2)	Tb1—O3—H3B	118.8 (16)
O3—Tb1—Cl1	143.22 (2)	H3A—O3—H3B	112 (2)
O3—Tb1—Cl1^i^	108.08 (2)		

Symmetry code: (i) −*x*+1, *y*, −*z*+1/2.

### Hexaaquadichloridoterbium(III) chloride (Tb) Hydrogen-bond geometry (Å, º)

**Table:**

*D*—H···*A*	*D*—H	H···*A*	*D*···*A*	*D*—H···*A*
O1—H1*A*···Cl1^ii^	0.77 (2)	2.41 (2)	3.1527 (9)	164 (2)
O1—H1*B*···Cl2^iii^	0.77 (2)	2.40 (2)	3.1661 (10)	172 (2)
O2—H2*A*···Cl1^iv^	0.82 (2)	2.33 (2)	3.1524 (10)	175 (2)
O2—H2*B*···Cl2^v^	0.76 (2)	2.51 (2)	3.2283 (10)	158.8 (19)
O3—H3*A*···Cl1^vi^	0.81 (2)	2.33 (2)	3.1297 (10)	173.2 (19)
O3—H3*B*···Cl2	0.77 (2)	2.43 (2)	3.1798 (9)	166 (2)

Symmetry codes: (ii) −*x*, −*y*, −*z*; (iii) −*x*, −*y*+1, −*z*; (iv) *x*, −*y*, *z*+1/2; (v) *x*, *y*−1, *z*; (vi) −*x*+1, *y*+1, −*z*+1/2.

### Hexaaquadichloridodysprosium(III) chloride (Dy) Crystal data

**Table:**

[DyCl_2_(H_2_O)_6_]Cl	*F*(000) = 354
*M**_r_* = 376.95	*D*_x_ = 2.586 Mg m^−^^3^
Monoclinic, *P*2/*c*	Ag *K*α radiation, λ = 0.56086 Å
*a* = 7.8439 (3) Å	Cell parameters from 9894 reflections
*b* = 6.4693 (3) Å	θ = 2.7–23.7°
*c* = 11.9660 (5) Å	µ = 4.51 mm^−^^1^
β = 127.143 (1)°	*T* = 100 K
*V* = 484.02 (4) Å^3^	Plate, yellow
*Z* = 2	0.23 × 0.19 × 0.14 mm

### Hexaaquadichloridodysprosium(III) chloride (Dy) Data collection

**Table:**

Bruker D8 Venture Duo diffractometer	1485 reflections with *I* > 2σ(*I*)
ω scans	*R*_int_ = 0.037
Absorption correction: multi-scan SADABS-2016/2 (Bruker, 2016)	θ_max_ = 23.8°, θ_min_ = 2.5°
*T*_min_ = 0.184, *T*_max_ = 0.257	*h* = −11→11
21844 measured reflections	*k* = −9→9
1501 independent reflections	*l* = −17→17

### Hexaaquadichloridodysprosium(III) chloride (Dy) Refinement

**Table:**

Refinement on *F*^2^	Hydrogen site location: difference Fourier map
Least-squares matrix: full	All H-atom parameters refined
*R*[*F*^2^ > 2σ(*F*^2^)] = 0.008	*w* = 1/[σ^2^(*F*_o_^2^) + (0.006*P*)^2^ + 0.0553*P*] where *P* = (*F*_o_^2^ + 2*F*_c_^2^)/3
*wR*(*F*^2^) = 0.018	(Δ/σ)_max_ < 0.001
*S* = 1.19	Δρ_max_ = 0.42 e Å^−^^3^
1501 reflections	Δρ_min_ = −0.48 e Å^−^^3^
72 parameters	Extinction correction: SHELXL-2018/1 (Sheldrick 2015b), Fc^*^=kFc[1+0.001xFc^2^λ^3^/sin(2θ)]^-1/4^
0 restraints	Extinction coefficient: 0.0098 (6)
Primary atom site location: dual	

### Hexaaquadichloridodysprosium(III) chloride (Dy) Special details

**Table:**

Geometry. All esds (except the esd in the dihedral angle between two l.s. planes) are estimated using the full covariance matrix. The cell esds are taken into account individually in the estimation of esds in distances, angles and torsion angles; correlations between esds in cell parameters are only used when they are defined by crystal symmetry. An approximate (isotropic) treatment of cell esds is used for estimating esds involving l.s. planes.

### Hexaaquadichloridodysprosium(III) chloride (Dy) Fractional atomic coordinates and isotropic or equivalent isotropic displacement parameters (Å^2^)

**Table:**

	*x*	*y*	*z*	*U*_iso_*/*U*_eq_	
Dy1	0.500000	0.15396 (2)	0.250000	0.00517 (3)	
Cl1	0.29906 (4)	−0.16263 (3)	0.05869 (3)	0.00928 (5)	
Cl2	0.000000	0.62489 (5)	0.250000	0.00990 (6)	
O1	0.16915 (13)	0.30103 (12)	0.06305 (8)	0.01025 (14)	
O2	0.23924 (13)	0.05125 (12)	0.28056 (9)	0.01049 (14)	
O3	0.44370 (14)	0.42381 (12)	0.35560 (8)	0.01037 (14)	
H1A	0.065 (3)	0.264 (3)	0.046 (2)	0.027 (4)*	
H1B	0.133 (3)	0.330 (2)	−0.011 (2)	0.030 (5)*	
H2A	0.253 (3)	0.086 (3)	0.349 (2)	0.034 (5)*	
H2B	0.195 (3)	−0.050 (3)	0.2644 (19)	0.026 (5)*	
H3A	0.513 (3)	0.525 (3)	0.3771 (18)	0.028 (4)*	
H3B	0.332 (3)	0.453 (3)	0.326 (2)	0.033 (5)*	

### Hexaaquadichloridodysprosium(III) chloride (Dy) Atomic displacement parameters (Å^2^)

**Table:**

	*U*^11^	*U*^22^	*U*^33^	*U*^12^	*U*^13^	*U*^23^
Dy1	0.00492 (4)	0.00544 (4)	0.00538 (4)	0.000	0.00322 (3)	0.000
Cl1	0.00897 (11)	0.00907 (10)	0.00889 (10)	−0.00078 (7)	0.00491 (9)	−0.00194 (7)
Cl2	0.00935 (15)	0.01086 (13)	0.01022 (15)	0.000	0.00630 (13)	0.000
O1	0.0068 (3)	0.0132 (3)	0.0090 (3)	0.0003 (3)	0.0038 (3)	0.0022 (3)
O2	0.0121 (4)	0.0100 (3)	0.0127 (4)	−0.0028 (3)	0.0093 (3)	−0.0018 (3)
O3	0.0084 (4)	0.0097 (3)	0.0137 (3)	−0.0014 (3)	0.0070 (3)	−0.0033 (3)

### Hexaaquadichloridodysprosium(III) chloride (Dy) Geometric parameters (Å, º)

**Table:**

Dy1—Cl1^i^	2.7500 (2)	Dy1—O3^i^	2.3480 (8)
Dy1—Cl1	2.7500 (2)	O1—H1A	0.755 (19)
Dy1—O1	2.3735 (8)	O1—H1B	0.78 (2)
Dy1—O1^i^	2.3735 (8)	O2—H2A	0.79 (2)
Dy1—O2^i^	2.3796 (8)	O2—H2B	0.712 (19)
Dy1—O2	2.3795 (8)	O3—H3A	0.787 (19)
Dy1—O3	2.3480 (8)	O3—H3B	0.75 (2)
			
Cl1—Dy1—Cl1^i^	83.719 (11)	O3^i^—Dy1—O1^i^	75.83 (3)
O1^i^—Dy1—Cl1^i^	76.29 (2)	O3—Dy1—O1^i^	69.43 (3)
O1—Dy1—Cl1	76.29 (2)	O3^i^—Dy1—O1	69.43 (3)
O1—Dy1—Cl1^i^	146.52 (2)	O3—Dy1—O1	75.83 (3)
O1^i^—Dy1—Cl1	146.52 (2)	O3—Dy1—O2	70.52 (3)
O1—Dy1—O1^i^	132.73 (4)	O3^i^—Dy1—O2^i^	70.52 (3)
O1^i^—Dy1—O2	121.28 (3)	O3^i^—Dy1—O2	138.48 (3)
O1^i^—Dy1—O2^i^	72.82 (3)	O3—Dy1—O2^i^	138.48 (3)
O1—Dy1—O2	72.82 (3)	O3^i^—Dy1—O3	83.94 (4)
O1—Dy1—O2^i^	121.28 (3)	Dy1—O1—H1A	121.1 (14)
O2—Dy1—Cl1	78.83 (2)	Dy1—O1—H1B	127.6 (15)
O2^i^—Dy1—Cl1^i^	78.82 (2)	H1A—O1—H1B	101 (2)
O2—Dy1—Cl1^i^	77.17 (2)	Dy1—O2—H2A	119.3 (15)
O2^i^—Dy1—Cl1	77.17 (2)	Dy1—O2—H2B	122.6 (15)
O2—Dy1—O2^i^	147.57 (4)	H2A—O2—H2B	106 (2)
O3—Dy1—Cl1	143.34 (2)	Dy1—O3—H3A	118.6 (13)
O3—Dy1—Cl1^i^	107.77 (2)	Dy1—O3—H3B	118.8 (15)
O3^i^—Dy1—Cl1	107.77 (2)	H3A—O3—H3B	109.0 (19)
O3^i^—Dy1—Cl1^i^	143.34 (2)		

Symmetry code: (i) −*x*+1, *y*, −*z*+1/2.

### Hexaaquadichloridodysprosium(III) chloride (Dy) Hydrogen-bond geometry (Å, º)

**Table:**

*D*—H···*A*	*D*—H	H···*A*	*D*···*A*	*D*—H···*A*
O1—H1*A*···Cl1^ii^	0.755 (19)	2.413 (19)	3.1545 (9)	167.6 (19)
O1—H1*B*···Cl2^iii^	0.78 (2)	2.39 (2)	3.1655 (8)	172.6 (16)
O2—H2*A*···Cl1^iv^	0.79 (2)	2.37 (2)	3.1511 (9)	175.7 (19)
O2—H2*B*···Cl2^v^	0.712 (19)	2.544 (19)	3.2303 (8)	162.7 (19)
O3—H3*A*···Cl1^vi^	0.787 (19)	2.342 (19)	3.1276 (8)	176.2 (18)
O3—H3*B*···Cl2	0.75 (2)	2.44 (2)	3.1766 (8)	167.1 (19)

Symmetry codes: (ii) −*x*, −*y*, −*z*; (iii) −*x*, −*y*+1, −*z*; (iv) *x*, −*y*, *z*+1/2; (v) *x*, *y*−1, *z*; (vi) −*x*+1, *y*+1, −*z*+1/2.

### Hexaaquadichloridoholmium(III) chloride (Ho) Crystal data

**Table:**

[HoCl_2_(H_2_O)_6_]Cl	*F*(000) = 356
*M**_r_* = 379.38	*D*_x_ = 2.611 Mg m^−^^3^
Monoclinic, *P*2/*c*	Ag *K*α radiation, λ = 0.56086 Å
*a* = 7.8303 (3) Å	Cell parameters from 7807 reflections
*b* = 6.4651 (2) Å	θ = 2.5–23.7°
*c* = 11.9509 (4) Å	µ = 4.77 mm^−^^1^
β = 127.086 (1)°	*T* = 100 K
*V* = 482.63 (3) Å^3^	Plate, yellow
*Z* = 2	0.14 × 0.10 × 0.07 mm

### Hexaaquadichloridoholmium(III) chloride (Ho) Data collection

**Table:**

Bruker D8 Venture Duo diffractometer	1454 reflections with *I* > 2σ(*I*)
ω scans	*R*_int_ = 0.026
Absorption correction: multi-scan SADABS-2016/2 (Bruker, 2016)	θ_max_ = 23.7°, θ_min_ = 2.5°
*T*_min_ = 0.222, *T*_max_ = 0.257	*h* = −11→11
10309 measured reflections	*k* = −9→9
1480 independent reflections	*l* = −17→17

### Hexaaquadichloridoholmium(III) chloride (Ho) Refinement

**Table:**

Refinement on *F*^2^	Hydrogen site location: difference Fourier map
Least-squares matrix: full	All H-atom parameters refined
*R*[*F*^2^ > 2σ(*F*^2^)] = 0.009	*w* = 1/[σ^2^(*F*_o_^2^) + (0.0056*P*)^2^ + 0.0614*P*] where *P* = (*F*_o_^2^ + 2*F*_c_^2^)/3
*wR*(*F*^2^) = 0.020	(Δ/σ)_max_ = 0.001
*S* = 1.07	Δρ_max_ = 0.31 e Å^−^^3^
1480 reflections	Δρ_min_ = −0.36 e Å^−^^3^
72 parameters	Extinction correction: SHELXL-2018/1 (Sheldrick 2015b), Fc^*^=kFc[1+0.001xFc^2^λ^3^/sin(2θ)]^-1/4^
0 restraints	Extinction coefficient: 0.0063 (5)
Primary atom site location: dual	

### Hexaaquadichloridoholmium(III) chloride (Ho) Special details

**Table:**

Geometry. All esds (except the esd in the dihedral angle between two l.s. planes) are estimated using the full covariance matrix. The cell esds are taken into account individually in the estimation of esds in distances, angles and torsion angles; correlations between esds in cell parameters are only used when they are defined by crystal symmetry. An approximate (isotropic) treatment of cell esds is used for estimating esds involving l.s. planes.

### Hexaaquadichloridoholmium(III) chloride (Ho) Fractional atomic coordinates and isotropic or equivalent isotropic displacement parameters (Å^2^)

**Table:**

	*x*	*y*	*z*	*U*_iso_*/*U*_eq_	
Ho1	0.500000	0.15471 (2)	0.250000	0.00548 (3)	
Cl1	0.29952 (5)	−0.16158 (4)	0.05941 (3)	0.00938 (5)	
Cl2	0.000000	0.62505 (6)	0.250000	0.01026 (8)	
O1	0.17011 (16)	0.30132 (14)	0.06373 (10)	0.01068 (17)	
O2	0.24000 (16)	0.05173 (14)	0.28059 (10)	0.01065 (17)	
O3	0.44395 (16)	0.42345 (14)	0.35534 (10)	0.01033 (17)	
H1A	0.063 (3)	0.262 (3)	0.047 (2)	0.027 (5)*	
H1B	0.140 (4)	0.330 (3)	−0.008 (3)	0.030 (6)*	
H2A	0.253 (4)	0.091 (3)	0.348 (2)	0.029 (6)*	
H2B	0.188 (3)	−0.059 (3)	0.260 (2)	0.026 (5)*	
H3A	0.518 (4)	0.522 (3)	0.384 (2)	0.035 (6)*	
H3B	0.322 (4)	0.459 (3)	0.325 (2)	0.029 (5)*	

### Hexaaquadichloridoholmium(III) chloride (Ho) Atomic displacement parameters (Å^2^)

**Table:**

	*U*^11^	*U*^22^	*U*^33^	*U*^12^	*U*^13^	*U*^23^
Ho1	0.00520 (4)	0.00572 (4)	0.00561 (4)	0.000	0.00329 (3)	0.000
Cl1	0.00892 (13)	0.00922 (11)	0.00888 (12)	−0.00084 (9)	0.00478 (11)	−0.00183 (9)
Cl2	0.00948 (18)	0.01124 (17)	0.01035 (18)	0.000	0.00613 (16)	0.000
O1	0.0081 (4)	0.0135 (4)	0.0091 (4)	0.0002 (3)	0.0045 (4)	0.0023 (3)
O2	0.0122 (4)	0.0098 (4)	0.0130 (4)	−0.0027 (3)	0.0092 (4)	−0.0023 (3)
O3	0.0085 (4)	0.0092 (4)	0.0135 (4)	−0.0011 (3)	0.0067 (4)	−0.0027 (3)

### Hexaaquadichloridoholmium(III) chloride (Ho) Geometric parameters (Å, º)

**Table:**

Ho1—Cl1^i^	2.7425 (3)	Ho1—O3^i^	2.3372 (9)
Ho1—Cl1	2.7425 (3)	O1—H1A	0.78 (2)
Ho1—O1	2.3646 (10)	O1—H1B	0.76 (2)
Ho1—O1^i^	2.3646 (10)	O2—H2A	0.79 (2)
Ho1—O2^i^	2.3706 (9)	O2—H2B	0.79 (2)
Ho1—O2	2.3705 (9)	O3—H3A	0.79 (2)
Ho1—O3	2.3372 (9)	O3—H3B	0.82 (2)
			
Cl1—Ho1—Cl1^i^	83.578 (12)	O3^i^—Ho1—O1^i^	75.86 (3)
O1^i^—Ho1—Cl1^i^	76.33 (2)	O3—Ho1—O1^i^	69.40 (3)
O1—Ho1—Cl1	76.33 (2)	O3^i^—Ho1—O1	69.41 (3)
O1—Ho1—Cl1^i^	146.51 (3)	O3—Ho1—O1	75.86 (3)
O1^i^—Ho1—Cl1	146.51 (3)	O3—Ho1—O2	70.61 (3)
O1—Ho1—O1^i^	132.74 (5)	O3^i^—Ho1—O2^i^	70.61 (3)
O1^i^—Ho1—O2	121.30 (3)	O3^i^—Ho1—O2	138.55 (3)
O1^i^—Ho1—O2^i^	72.88 (3)	O3—Ho1—O2^i^	138.55 (3)
O1—Ho1—O2	72.88 (3)	O3^i^—Ho1—O3	83.96 (5)
O1—Ho1—O2^i^	121.31 (3)	Ho1—O1—H1A	120.3 (16)
O2—Ho1—Cl1	78.70 (2)	Ho1—O1—H1B	125.2 (17)
O2^i^—Ho1—Cl1^i^	78.70 (2)	H1A—O1—H1B	104 (2)
O2—Ho1—Cl1^i^	77.13 (2)	Ho1—O2—H2A	118.2 (16)
O2^i^—Ho1—Cl1	77.13 (2)	Ho1—O2—H2B	122.9 (15)
O2—Ho1—O2^i^	147.38 (5)	H2A—O2—H2B	109 (2)
O3—Ho1—Cl1	143.35 (3)	Ho1—O3—H3A	120.6 (16)
O3—Ho1—Cl1^i^	107.83 (2)	Ho1—O3—H3B	121.1 (14)
O3^i^—Ho1—Cl1	107.83 (2)	H3A—O3—H3B	109 (2)
O3^i^—Ho1—Cl1^i^	143.35 (3)		

Symmetry code: (i) −*x*+1, *y*, −*z*+1/2.

### Hexaaquadichloridoholmium(III) chloride (Ho) Hydrogen-bond geometry (Å, º)

**Table:**

*D*—H···*A*	*D*—H	H···*A*	*D*···*A*	*D*—H···*A*
O1—H1*A*···Cl1^ii^	0.78 (2)	2.40 (2)	3.1589 (11)	166 (2)
O1—H1*B*···Cl2^iii^	0.76 (2)	2.41 (2)	3.1678 (10)	170 (2)
O2—H2*A*···Cl1^iv^	0.79 (2)	2.37 (2)	3.1546 (10)	172 (2)
O2—H2*B*···Cl2^v^	0.79 (2)	2.48 (2)	3.2320 (10)	161.6 (19)
O3—H3*A*···Cl1^vi^	0.79 (2)	2.35 (2)	3.1309 (10)	172 (2)
O3—H3*B*···Cl2	0.82 (2)	2.36 (2)	3.1765 (10)	168.9 (19)

Symmetry codes: (ii) −*x*, −*y*, −*z*; (iii) −*x*, −*y*+1, −*z*; (iv) *x*, −*y*, *z*+1/2; (v) *x*, *y*−1, *z*; (vi) −*x*+1, *y*+1, −*z*+1/2.

### Hexaaquadichloridoerbium(III) chloride (Er) Crystal data

**Table:**

[ErCl_2_(H_2_O)_6_]Cl	*F*(000) = 358
*M**_r_* = 381.71	*D*_x_ = 2.648 Mg m^−^^3^
Monoclinic, *P*2/*c*	Ag *K*α radiation, λ = 0.56086 Å
*a* = 7.8035 (2) Å	Cell parameters from 9658 reflections
*b* = 6.4488 (2) Å	θ = 2.5–23.7°
*c* = 11.9182 (4) Å	µ = 5.07 mm^−^^1^
β = 127.044 (1)°	*T* = 100 K
*V* = 478.71 (3) Å^3^	Plate, pink
*Z* = 2	0.23 × 0.22 × 0.17 mm

### Hexaaquadichloridoerbium(III) chloride (Er) Data collection

**Table:**

Bruker D8 Venture Duo diffractometer	1479 reflections with *I* > 2σ(*I*)
ω scans	*R*_int_ = 0.034
Absorption correction: multi-scan SADABS-2016/2 (Bruker, 2016)	θ_max_ = 23.7°, θ_min_ = 2.5°
*T*_min_ = 0.175, *T*_max_ = 0.257	*h* = −11→10
24599 measured reflections	*k* = −9→9
1481 independent reflections	*l* = −17→17

### Hexaaquadichloridoerbium(III) chloride (Er) Refinement

**Table:**

Refinement on *F*^2^	Hydrogen site location: difference Fourier map
Least-squares matrix: full	All H-atom parameters refined
*R*[*F*^2^ > 2σ(*F*^2^)] = 0.009	*w* = 1/[σ^2^(*F*_o_^2^) + (0.0069*P*)^2^ + 0.1191*P*] where *P* = (*F*_o_^2^ + 2*F*_c_^2^)/3
*wR*(*F*^2^) = 0.020	(Δ/σ)_max_ = 0.001
*S* = 1.30	Δρ_max_ = 0.47 e Å^−^^3^
1481 reflections	Δρ_min_ = −0.88 e Å^−^^3^
72 parameters	Extinction correction: SHELXL-2018/1 (Sheldrick 2015b), Fc^*^=kFc[1+0.001xFc^2^λ^3^/sin(2θ)]^-1/4^
0 restraints	Extinction coefficient: 0.0321 (9)
Primary atom site location: structure-invariant direct methods	

### Hexaaquadichloridoerbium(III) chloride (Er) Special details

**Table:**

Geometry. All esds (except the esd in the dihedral angle between two l.s. planes) are estimated using the full covariance matrix. The cell esds are taken into account individually in the estimation of esds in distances, angles and torsion angles; correlations between esds in cell parameters are only used when they are defined by crystal symmetry. An approximate (isotropic) treatment of cell esds is used for estimating esds involving l.s. planes.

### Hexaaquadichloridoerbium(III) chloride (Er) Fractional atomic coordinates and isotropic or equivalent isotropic displacement parameters (Å^2^)

**Table:**

	*x*	*y*	*z*	*U*_iso_*/*U*_eq_	
Er1	0.500000	0.15555 (2)	0.250000	0.00519 (3)	
Cl1	0.29999 (4)	−0.16033 (4)	0.05990 (3)	0.00919 (5)	
Cl2	0.000000	0.62527 (6)	0.250000	0.00995 (7)	
O1	0.17058 (14)	0.30150 (14)	0.06411 (10)	0.01031 (15)	
O2	0.24065 (14)	0.05247 (15)	0.28058 (10)	0.01036 (15)	
O3	0.44402 (15)	0.42330 (14)	0.35511 (10)	0.01044 (15)	
H1A	0.062 (4)	0.263 (4)	0.048 (2)	0.029 (5)*	
H1B	0.141 (4)	0.332 (3)	−0.007 (3)	0.029 (6)*	
H2A	0.259 (4)	0.083 (4)	0.348 (2)	0.027 (5)*	
H2B	0.193 (3)	−0.061 (4)	0.263 (2)	0.024 (5)*	
H3A	0.518 (4)	0.519 (4)	0.380 (2)	0.029 (5)*	
H3B	0.327 (4)	0.455 (4)	0.323 (2)	0.030 (5)*	

### Hexaaquadichloridoerbium(III) chloride (Er) Atomic displacement parameters (Å^2^)

**Table:**

	*U*^11^	*U*^22^	*U*^33^	*U*^12^	*U*^13^	*U*^23^
Er1	0.00491 (4)	0.00568 (4)	0.00476 (4)	0.000	0.00279 (3)	0.000
Cl1	0.00883 (11)	0.00906 (12)	0.00820 (12)	−0.00080 (8)	0.00435 (10)	−0.00190 (8)
Cl2	0.00931 (15)	0.01105 (16)	0.00951 (16)	0.000	0.00568 (14)	0.000
O1	0.0075 (4)	0.0128 (4)	0.0083 (4)	0.0003 (3)	0.0036 (3)	0.0022 (3)
O2	0.0119 (4)	0.0105 (4)	0.0110 (4)	−0.0024 (3)	0.0081 (3)	−0.0017 (3)
O3	0.0087 (4)	0.0091 (4)	0.0130 (4)	−0.0011 (3)	0.0063 (3)	−0.0033 (3)

### Hexaaquadichloridoerbium(III) chloride (Er) Geometric parameters (Å, º)

**Table:**

Er1—Cl1	2.7309 (3)	Er1—O3^i^	2.3239 (9)
Er1—Cl1^i^	2.7309 (3)	O1—H1A	0.78 (2)
Er1—O1	2.3538 (9)	O1—H1B	0.76 (3)
Er1—O1^i^	2.3538 (9)	O2—H2A	0.76 (2)
Er1—O2^i^	2.3579 (9)	O2—H2B	0.79 (2)
Er1—O2	2.3578 (9)	O3—H3A	0.77 (2)
Er1—O3	2.3239 (9)	O3—H3B	0.78 (2)
			
Cl1^i^—Er1—Cl1	83.525 (12)	O3^i^—Er1—O1^i^	75.93 (3)
O1^i^—Er1—Cl1	146.45 (2)	O3—Er1—O1^i^	69.45 (3)
O1—Er1—Cl1^i^	146.45 (2)	O3^i^—Er1—O1	69.45 (3)
O1—Er1—Cl1	76.30 (2)	O3—Er1—O1	75.93 (3)
O1^i^—Er1—Cl1^i^	76.30 (2)	O3—Er1—O2	70.65 (3)
O1—Er1—O1^i^	132.86 (5)	O3^i^—Er1—O2^i^	70.65 (3)
O1^i^—Er1—O2	121.31 (3)	O3^i^—Er1—O2	138.62 (3)
O1^i^—Er1—O2^i^	72.89 (3)	O3—Er1—O2^i^	138.62 (3)
O1—Er1—O2	72.89 (3)	O3^i^—Er1—O3	84.03 (5)
O1—Er1—O2^i^	121.31 (3)	Er1—O1—H1A	120.7 (17)
O2—Er1—Cl1^i^	77.07 (2)	Er1—O1—H1B	125.5 (19)
O2^i^—Er1—Cl1	77.07 (2)	H1A—O1—H1B	104 (2)
O2—Er1—Cl1	78.64 (2)	Er1—O2—H2A	117.7 (17)
O2^i^—Er1—Cl1^i^	78.64 (2)	Er1—O2—H2B	122.2 (15)
O2—Er1—O2^i^	147.25 (5)	H2A—O2—H2B	106 (2)
O3—Er1—Cl1^i^	107.82 (2)	Er1—O3—H3A	117.0 (17)
O3—Er1—Cl1	143.35 (2)	Er1—O3—H3B	118.2 (17)
O3^i^—Er1—Cl1^i^	143.35 (2)	H3A—O3—H3B	112 (2)
O3^i^—Er1—Cl1	107.82 (2)		

Symmetry code: (i) −*x*+1, *y*, −*z*+1/2.

### Hexaaquadichloridoerbium(III) chloride (Er) Hydrogen-bond geometry (Å, º)

**Table:**

*D*—H···*A*	*D*—H	H···*A*	*D*···*A*	*D*—H···*A*
O1—H1*A*···Cl1^ii^	0.78 (2)	2.39 (2)	3.1557 (10)	166 (2)
O1—H1*B*···Cl2^iii^	0.76 (3)	2.41 (3)	3.1632 (10)	170 (2)
O2—H2*A*···Cl1^iv^	0.76 (2)	2.39 (2)	3.1502 (10)	176 (2)
O2—H2*B*···Cl2^v^	0.79 (2)	2.47 (2)	3.2287 (10)	161 (2)
O3—H3*A*···Cl1^vi^	0.77 (2)	2.36 (2)	3.1286 (10)	172 (2)
O3—H3*B*···Cl2	0.78 (2)	2.41 (2)	3.1689 (9)	167 (2)

Symmetry codes: (ii) −*x*, −*y*, −*z*; (iii) −*x*, −*y*+1, −*z*; (iv) *x*, −*y*, *z*+1/2; (v) *x*, *y*−1, *z*; (vi) −*x*+1, *y*+1, −*z*+1/2.

### Hexaaquadichloridotantalum(III) chloride (Tm) Crystal data

**Table:**

[TmCl_2_(H_2_O)_6_]Cl	*F*(000) = 360
*M**_r_* = 383.38	*D*_x_ = 2.671 Mg m^−^^3^
Monoclinic, *P*2/*c*	Ag *K*α radiation, λ = 0.56086 Å
*a* = 7.7889 (4) Å	Cell parameters from 9923 reflections
*b* = 6.4490 (3) Å	θ = 2.6–23.7°
*c* = 11.8760 (6) Å	µ = 5.37 mm^−^^1^
β = 126.961 (1)°	*T* = 100 K
*V* = 476.66 (4) Å^3^	Plate, colorless
*Z* = 2	0.17 × 0.11 × 0.10 mm

### Hexaaquadichloridotantalum(III) chloride (Tm) Data collection

**Table:**

Bruker D8 Venture Duo diffractometer	1464 reflections with *I* > 2σ(*I*)
ω scans	*R*_int_ = 0.033
Absorption correction: multi-scan SADABS-2016/2 (Bruker, 2016)	θ_max_ = 23.7°, θ_min_ = 2.5°
*T*_min_ = 0.207, *T*_max_ = 0.257	*h* = −11→11
16364 measured reflections	*k* = −9→9
1481 independent reflections	*l* = −17→16

### Hexaaquadichloridotantalum(III) chloride (Tm) Refinement

**Table:**

Refinement on *F*^2^	Hydrogen site location: difference Fourier map
Least-squares matrix: full	All H-atom parameters refined
*R*[*F*^2^ > 2σ(*F*^2^)] = 0.007	*w* = 1/[σ^2^(*F*_o_^2^) + 0.0685*P*] where *P* = (*F*_o_^2^ + 2*F*_c_^2^)/3
*wR*(*F*^2^) = 0.016	(Δ/σ)_max_ = 0.001
*S* = 1.15	Δρ_max_ = 0.39 e Å^−^^3^
1481 reflections	Δρ_min_ = −0.48 e Å^−^^3^
72 parameters	Extinction correction: SHELXL-2018/1 (Sheldrick 2015b), Fc^*^=kFc[1+0.001xFc^2^λ^3^/sin(2θ)]^-1/4^
0 restraints	Extinction coefficient: 0.0056 (5)
Primary atom site location: dual	

### Hexaaquadichloridotantalum(III) chloride (Tm) Special details

**Table:**

Geometry. All esds (except the esd in the dihedral angle between two l.s. planes) are estimated using the full covariance matrix. The cell esds are taken into account individually in the estimation of esds in distances, angles and torsion angles; correlations between esds in cell parameters are only used when they are defined by crystal symmetry. An approximate (isotropic) treatment of cell esds is used for estimating esds involving l.s. planes.

### Hexaaquadichloridotantalum(III) chloride (Tm) Fractional atomic coordinates and isotropic or equivalent isotropic displacement parameters (Å^2^)

**Table:**

	*x*	*y*	*z*	*U*_iso_*/*U*_eq_	
Tm1	0.500000	0.15657 (2)	0.250000	0.00505 (3)	
Cl1	0.30008 (4)	−0.15947 (3)	0.06069 (2)	0.00896 (4)	
Cl2	0.000000	0.62678 (5)	0.250000	0.00941 (6)	
O1	0.17163 (12)	0.30137 (11)	0.06506 (8)	0.00987 (13)	
O2	0.24195 (12)	0.05360 (12)	0.28076 (8)	0.00989 (13)	
O3	0.44427 (13)	0.42399 (11)	0.35464 (8)	0.01000 (13)	
H1A	0.072 (3)	0.261 (3)	0.050 (2)	0.032 (5)*	
H1B	0.140 (3)	0.331 (2)	−0.012 (2)	0.020 (4)*	
H2A	0.252 (3)	0.089 (3)	0.347 (2)	0.033 (5)*	
H2B	0.197 (3)	−0.052 (3)	0.2624 (18)	0.019 (4)*	
H3A	0.518 (3)	0.523 (3)	0.3800 (19)	0.028 (4)*	
H3B	0.332 (3)	0.458 (3)	0.3247 (19)	0.023 (4)*	

### Hexaaquadichloridotantalum(III) chloride (Tm) Atomic displacement parameters (Å^2^)

**Table:**

	*U*^11^	*U*^22^	*U*^33^	*U*^12^	*U*^13^	*U*^23^
Tm1	0.00468 (3)	0.00528 (3)	0.00526 (3)	0.000	0.00302 (2)	0.000
Cl1	0.00875 (9)	0.00886 (9)	0.00844 (9)	−0.00085 (7)	0.00472 (8)	−0.00185 (7)
Cl2	0.00877 (13)	0.01055 (13)	0.00955 (13)	0.000	0.00584 (12)	0.000
O1	0.0068 (3)	0.0126 (3)	0.0084 (3)	0.0002 (2)	0.0037 (3)	0.0021 (2)
O2	0.0117 (3)	0.0094 (3)	0.0117 (3)	−0.0028 (3)	0.0087 (3)	−0.0021 (3)
O3	0.0085 (3)	0.0087 (3)	0.0134 (3)	−0.0013 (3)	0.0069 (3)	−0.0033 (2)

### Hexaaquadichloridotantalum(III) chloride (Tm) Geometric parameters (Å, º)

**Table:**

Tm1—Cl1	2.7246 (2)	Tm1—O3^i^	2.3142 (7)
Tm1—Cl1^i^	2.7246 (2)	O1—H1A	0.73 (2)
Tm1—O1^i^	2.3414 (7)	O1—H1B	0.81 (2)
Tm1—O1	2.3414 (7)	O2—H2A	0.78 (2)
Tm1—O2^i^	2.3446 (8)	O2—H2B	0.737 (18)
Tm1—O2	2.3446 (8)	O3—H3A	0.789 (19)
Tm1—O3	2.3142 (7)	O3—H3B	0.75 (2)
			
Cl1^i^—Tm1—Cl1	83.158 (10)	O3^i^—Tm1—O1	69.57 (3)
O1—Tm1—Cl1	76.368 (19)	O3—Tm1—O1	75.80 (3)
O1^i^—Tm1—Cl1^i^	76.368 (19)	O3^i^—Tm1—O1^i^	75.80 (3)
O1^i^—Tm1—Cl1	146.34 (2)	O3—Tm1—O1^i^	69.57 (3)
O1—Tm1—Cl1^i^	146.34 (2)	O3—Tm1—O2	70.87 (3)
O1^i^—Tm1—O1	132.99 (4)	O3^i^—Tm1—O2^i^	70.87 (3)
O1—Tm1—O2	72.77 (3)	O3^i^—Tm1—O2	138.58 (3)
O1—Tm1—O2^i^	121.47 (3)	O3—Tm1—O2^i^	138.58 (3)
O1^i^—Tm1—O2	121.47 (3)	O3^i^—Tm1—O3	83.65 (4)
O1^i^—Tm1—O2^i^	72.77 (3)	Tm1—O1—H1A	118.9 (16)
O2—Tm1—Cl1^i^	77.157 (19)	Tm1—O1—H1B	124.8 (13)
O2^i^—Tm1—Cl1	77.156 (19)	H1A—O1—H1B	105 (2)
O2—Tm1—Cl1	78.38 (2)	Tm1—O2—H2A	120.0 (15)
O2^i^—Tm1—Cl1^i^	78.38 (2)	Tm1—O2—H2B	120.9 (14)
O2—Tm1—O2^i^	147.09 (4)	H2A—O2—H2B	108 (2)
O3—Tm1—Cl1^i^	108.24 (2)	Tm1—O3—H3A	118.8 (14)
O3—Tm1—Cl1	143.31 (2)	Tm1—O3—H3B	120.2 (13)
O3^i^—Tm1—Cl1^i^	143.31 (2)	H3A—O3—H3B	108.5 (19)
O3^i^—Tm1—Cl1	108.24 (2)		

Symmetry code: (i) −*x*+1, *y*, −*z*+1/2.

### Hexaaquadichloridotantalum(III) chloride (Tm) Hydrogen-bond geometry (Å, º)

**Table:**

*D*—H···*A*	*D*—H	H···*A*	*D*···*A*	*D*—H···*A*
O1—H1*A*···Cl1^ii^	0.73 (2)	2.44 (2)	3.1573 (8)	165 (2)
O1—H1*B*···Cl2^iii^	0.81 (2)	2.36 (2)	3.1602 (8)	169.7 (16)
O2—H2*A*···Cl1^iv^	0.78 (2)	2.38 (2)	3.1485 (8)	173.4 (19)
O2—H2*B*···Cl2^v^	0.737 (18)	2.528 (18)	3.2301 (8)	159.7 (18)
O3—H3*A*···Cl1^vi^	0.789 (19)	2.343 (19)	3.1280 (8)	173.4 (18)
O3—H3*B*···Cl2	0.75 (2)	2.43 (2)	3.1714 (8)	169.4 (17)

Symmetry codes: (ii) −*x*, −*y*, −*z*; (iii) −*x*, −*y*+1, −*z*; (iv) *x*, −*y*, *z*+1/2; (v) *x*, *y*−1, *z*; (vi) −*x*+1, *y*+1, −*z*+1/2.

### Hexaaquadichloridoytterbium(III) chloride (Yb) Crystal data

**Table:**

[YbCl_2_(H_2_O)_6_]Cl	*F*(000) = 362
*M**_r_* = 387.49	*D*_x_ = 2.717 Mg m^−^^3^
Monoclinic, *P*2/*c*	Ag *K*α radiation, λ = 0.56086 Å
*a* = 7.7666 (2) Å	Cell parameters from 9892 reflections
*b* = 6.4305 (2) Å	θ = 3.0–23.7°
*c* = 11.8745 (3) Å	µ = 5.69 mm^−^^1^
β = 126.991 (1)°	*T* = 100 K
*V* = 473.69 (2) Å^3^	Plate, colorless
*Z* = 2	0.30 × 0.23 × 0.20 mm

### Hexaaquadichloridoytterbium(III) chloride (Yb) Data collection

**Table:**

Bruker D8 Venture Duo diffractometer	1439 reflections with *I* > 2σ(*I*)
ω scans	*R*_int_ = 0.035
Absorption correction: multi-scan SADABS-2016/2 (Bruker, 2016)	θ_max_ = 23.7°, θ_min_ = 2.5°
*T*_min_ = 0.168, *T*_max_ = 0.257	*h* = −11→11
19224 measured reflections	*k* = −9→9
1464 independent reflections	*l* = −16→16

### Hexaaquadichloridoytterbium(III) chloride (Yb) Refinement

**Table:**

Refinement on *F*^2^	Hydrogen site location: difference Fourier map
Least-squares matrix: full	All H-atom parameters refined
*R*[*F*^2^ > 2σ(*F*^2^)] = 0.008	*w* = 1/[σ^2^(*F*_o_^2^) + (0.0064*P*)^2^] where *P* = (*F*_o_^2^ + 2*F*_c_^2^)/3
*wR*(*F*^2^) = 0.017	(Δ/σ)_max_ = 0.001
*S* = 1.19	Δρ_max_ = 0.62 e Å^−^^3^
1464 reflections	Δρ_min_ = −0.74 e Å^−^^3^
72 parameters	Extinction correction: SHELXL-2018/1 (Sheldrick 2015b), Fc^*^=kFc[1+0.001xFc^2^λ^3^/sin(2θ)]^-1/4^
0 restraints	Extinction coefficient: 0.0290 (7)
Primary atom site location: dual	

### Hexaaquadichloridoytterbium(III) chloride (Yb) Special details

**Table:**

Geometry. All esds (except the esd in the dihedral angle between two l.s. planes) are estimated using the full covariance matrix. The cell esds are taken into account individually in the estimation of esds in distances, angles and torsion angles; correlations between esds in cell parameters are only used when they are defined by crystal symmetry. An approximate (isotropic) treatment of cell esds is used for estimating esds involving l.s. planes.

### Hexaaquadichloridoytterbium(III) chloride (Yb) Fractional atomic coordinates and isotropic or equivalent isotropic displacement parameters (Å^2^)

**Table:**

	*x*	*y*	*z*	*U*_iso_*/*U*_eq_	
Yb1	0.500000	0.15772 (2)	0.250000	0.00506 (3)	
Cl1	0.30072 (4)	−0.15810 (3)	0.06078 (3)	0.00886 (5)	
Cl2	0.000000	0.62670 (5)	0.250000	0.00966 (7)	
O1	0.17225 (14)	0.30159 (13)	0.06541 (9)	0.01013 (15)	
O2	0.24258 (13)	0.05444 (13)	0.28053 (9)	0.00973 (15)	
O3	0.44456 (14)	0.42323 (12)	0.35473 (9)	0.01004 (15)	
H1A	0.069 (3)	0.261 (3)	0.047 (2)	0.030 (5)*	
H1B	0.148 (3)	0.330 (3)	−0.003 (2)	0.025 (5)*	
H2A	0.257 (3)	0.085 (3)	0.344 (2)	0.028 (5)*	
H2B	0.193 (3)	−0.059 (3)	0.2600 (19)	0.022 (4)*	
H3A	0.509 (3)	0.516 (3)	0.3768 (19)	0.025 (5)*	
H3B	0.318 (3)	0.461 (3)	0.321 (2)	0.026 (4)*	

### Hexaaquadichloridoytterbium(III) chloride (Yb) Atomic displacement parameters (Å^2^)

**Table:**

	*U*^11^	*U*^22^	*U*^33^	*U*^12^	*U*^13^	*U*^23^
Yb1	0.00471 (4)	0.00511 (4)	0.00532 (4)	0.000	0.00301 (3)	0.000
Cl1	0.00835 (11)	0.00860 (10)	0.00832 (12)	−0.00077 (8)	0.00433 (10)	−0.00180 (8)
Cl2	0.00878 (15)	0.01064 (14)	0.00987 (17)	0.000	0.00577 (14)	0.000
O1	0.0073 (4)	0.0127 (3)	0.0087 (4)	0.0002 (3)	0.0040 (3)	0.0025 (3)
O2	0.0110 (4)	0.0096 (3)	0.0111 (4)	−0.0024 (3)	0.0079 (3)	−0.0020 (3)
O3	0.0084 (3)	0.0081 (3)	0.0137 (4)	−0.0015 (3)	0.0066 (3)	−0.0030 (3)

### Hexaaquadichloridoytterbium(III) chloride (Yb) Geometric parameters (Å, º)

**Table:**

Yb1—Cl1	2.7173 (3)	Yb1—H2A	2.74 (2)
Yb1—Cl1^i^	2.7173 (3)	Yb1—H3A	2.729 (18)
Yb1—O1	2.3293 (8)	O1—H1A	0.74 (2)
Yb1—O1^i^	2.3293 (8)	O1—H1B	0.74 (2)
Yb1—O2^i^	2.3329 (8)	O2—H2A	0.72 (2)
Yb1—O2	2.3328 (8)	O2—H2B	0.795 (19)
Yb1—O3^i^	2.3003 (8)	O3—H3A	0.720 (19)
Yb1—O3	2.3003 (8)	O3—H3B	0.84 (2)
			
Cl1^i^—Yb1—Cl1	83.270 (11)	O3^i^—Yb1—Cl1	107.85 (2)
Cl1^i^—Yb1—H2A	74.1 (4)	O3^i^—Yb1—Cl1^i^	143.42 (2)
Cl1—Yb1—H2A	91.1 (4)	O3—Yb1—Cl1^i^	107.85 (2)
Cl1—Yb1—H3A	154.1 (4)	O3—Yb1—Cl1	143.42 (2)
Cl1^i^—Yb1—H3A	111.2 (4)	O3^i^—Yb1—O1^i^	76.11 (3)
O1^i^—Yb1—Cl1^i^	76.26 (2)	O3—Yb1—O1^i^	69.54 (3)
O1—Yb1—Cl1^i^	146.22 (2)	O3^i^—Yb1—O1	69.55 (3)
O1^i^—Yb1—Cl1	146.22 (2)	O3—Yb1—O1	76.11 (3)
O1—Yb1—Cl1	76.26 (2)	O3—Yb1—O2^i^	138.77 (3)
O1—Yb1—O1^i^	133.20 (4)	O3—Yb1—O2	70.77 (3)
O1—Yb1—O2^i^	121.35 (3)	O3^i^—Yb1—O2^i^	70.77 (3)
O1—Yb1—O2	72.89 (3)	O3^i^—Yb1—O2	138.77 (3)
O1^i^—Yb1—O2	121.35 (3)	O3—Yb1—O3^i^	84.16 (4)
O1^i^—Yb1—O2^i^	72.89 (3)	O3—Yb1—H2A	60.6 (4)
O1^i^—Yb1—H2A	108.3 (4)	O3^i^—Yb1—H2A	138.0 (4)
O1—Yb1—H2A	79.7 (4)	O3—Yb1—H3A	13.3 (4)
O1—Yb1—H3A	80.5 (4)	O3^i^—Yb1—H3A	73.8 (4)
O1^i^—Yb1—H3A	59.6 (4)	H2A—Yb1—H3A	73.5 (6)
O2^i^—Yb1—Cl1^i^	78.50 (2)	Yb1—O1—H1A	120.9 (16)
O2—Yb1—Cl1	78.50 (2)	Yb1—O1—H1B	122.8 (16)
O2—Yb1—Cl1^i^	76.92 (2)	H1A—O1—H1B	105 (2)
O2^i^—Yb1—Cl1	76.92 (2)	Yb1—O2—H2A	118.2 (16)
O2—Yb1—O2^i^	146.92 (4)	Yb1—O2—H2B	121.4 (12)
O2^i^—Yb1—H2A	151.2 (4)	H2A—O2—H2B	108 (2)
O2—Yb1—H2A	13.3 (4)	Yb1—O3—H3A	119.6 (14)
O2—Yb1—H3A	84.0 (4)	Yb1—O3—H3B	120.0 (13)
O2^i^—Yb1—H3A	125.8 (4)	H3A—O3—H3B	107.0 (18)

Symmetry code: (i) −*x*+1, *y*, −*z*+1/2.

### Hexaaquadichloridoytterbium(III) chloride (Yb) Hydrogen-bond geometry (Å, º)

**Table:**

*D*—H···*A*	*D*—H	H···*A*	*D*···*A*	*D*—H···*A*
O1—H1*A*···Cl1^ii^	0.74 (2)	2.43 (2)	3.1570 (9)	168 (2)
O1—H1*B*···Cl2^iii^	0.74 (2)	2.44 (2)	3.1623 (9)	167 (2)
O2—H2*A*···Cl1^iv^	0.72 (2)	2.44 (2)	3.1486 (9)	175 (2)
O2—H2*B*···Cl2^v^	0.795 (19)	2.472 (19)	3.2287 (9)	159.5 (16)
O3—H3*A*···Cl1^vi^	0.720 (19)	2.409 (19)	3.1273 (9)	175.4 (19)
O3—H3*B*···Cl2	0.84 (2)	2.33 (2)	3.1643 (8)	168.8 (17)

Symmetry codes: (ii) −*x*, −*y*, −*z*; (iii) −*x*, −*y*+1, −*z*; (iv) *x*, −*y*, *z*+1/2; (v) *x*, *y*−1, *z*; (vi) −*x*+1, *y*+1, −*z*+1/2.

### Hexaaquadichloridolutetium(III) chloride (Lu) Crystal data

**Table:**

[LuCl_2_(H_2_O)_6_]Cl	*F*(000) = 364
*M**_r_* = 389.42	*D*_x_ = 2.737 Mg m^−^^3^
Monoclinic, *P*2/*c*	Ag *K*α radiation, λ = 0.56086 Å
*a* = 7.7621 (3) Å	Cell parameters from 9948 reflections
*b* = 6.4241 (3) Å	θ = 2.5–23.7°
*c* = 11.8671 (5) Å	µ = 6.00 mm^−^^1^
β = 127.008 (1)°	*T* = 100 K
*V* = 472.54 (4) Å^3^	Plate, colorless
*Z* = 2	0.17 × 0.14 × 0.14 mm

### Hexaaquadichloridolutetium(III) chloride (Lu) Data collection

**Table:**

Bruker D8 Venture Duo diffractometer	1436 reflections with *I* > 2σ(*I*)
ω scans	*R*_int_ = 0.041
Absorption correction: multi-scan SADABS-2016/2 (Bruker, 2016)	θ_max_ = 23.7°, θ_min_ = 2.5°
*T*_min_ = 0.201, *T*_max_ = 0.257	*h* = −11→11
23799 measured reflections	*k* = −9→9
1464 independent reflections	*l* = −16→17

### Hexaaquadichloridolutetium(III) chloride (Lu) Refinement

**Table:**

Refinement on *F*^2^	Hydrogen site location: difference Fourier map
Least-squares matrix: full	All H-atom parameters refined
*R*[*F*^2^ > 2σ(*F*^2^)] = 0.009	*w* = 1/[σ^2^(*F*_o_^2^) + 0.3367*P*] where *P* = (*F*_o_^2^ + 2*F*_c_^2^)/3
*wR*(*F*^2^) = 0.020	(Δ/σ)_max_ < 0.001
*S* = 1.28	Δρ_max_ = 0.78 e Å^−^^3^
1464 reflections	Δρ_min_ = −0.82 e Å^−^^3^
72 parameters	Extinction correction: SHELXL-2018/1 (Sheldrick 2015b), Fc^*^=kFc[1+0.001xFc^2^λ^3^/sin(2θ)]^-1/4^
0 restraints	Extinction coefficient: 0.0094 (5)
Primary atom site location: dual	

### Hexaaquadichloridolutetium(III) chloride (Lu) Special details

**Table:**

Geometry. All esds (except the esd in the dihedral angle between two l.s. planes) are estimated using the full covariance matrix. The cell esds are taken into account individually in the estimation of esds in distances, angles and torsion angles; correlations between esds in cell parameters are only used when they are defined by crystal symmetry. An approximate (isotropic) treatment of cell esds is used for estimating esds involving l.s. planes.

### Hexaaquadichloridolutetium(III) chloride (Lu) Fractional atomic coordinates and isotropic or equivalent isotropic displacement parameters (Å^2^)

**Table:**

	*x*	*y*	*z*	*U*_iso_*/*U*_eq_	
Lu1	0.500000	0.15824 (2)	0.250000	0.00529 (4)	
Cl1	0.30110 (5)	−0.15719 (6)	0.06103 (4)	0.00912 (6)	
Cl2	0.000000	0.62590 (8)	0.250000	0.00994 (9)	
O1	0.17244 (18)	0.30166 (18)	0.06562 (12)	0.0104 (2)	
O2	0.24293 (19)	0.05447 (19)	0.28021 (12)	0.0101 (2)	
O3	0.4452 (2)	0.42271 (19)	0.35486 (12)	0.0102 (2)	
H1A	0.070 (4)	0.258 (4)	0.052 (3)	0.027 (7)*	
H1B	0.142 (4)	0.328 (4)	−0.007 (3)	0.022 (6)*	
H2A	0.255 (4)	0.086 (4)	0.344 (3)	0.028 (7)*	
H2B	0.193 (4)	−0.064 (5)	0.261 (3)	0.033 (7)*	
H3A	0.517 (5)	0.523 (5)	0.378 (3)	0.037 (8)*	
H3B	0.328 (4)	0.454 (4)	0.322 (3)	0.026 (7)*	

### Hexaaquadichloridolutetium(III) chloride (Lu) Atomic displacement parameters (Å^2^)

**Table:**

	*U*^11^	*U*^22^	*U*^33^	*U*^12^	*U*^13^	*U*^23^
Lu1	0.00480 (5)	0.00564 (5)	0.00535 (4)	0.000	0.00301 (3)	0.000
Cl1	0.00841 (14)	0.00916 (14)	0.00864 (14)	−0.00059 (12)	0.00451 (12)	−0.00171 (12)
Cl2	0.0089 (2)	0.0111 (2)	0.0103 (2)	0.000	0.00602 (18)	0.000
O1	0.0070 (5)	0.0131 (5)	0.0093 (5)	−0.0002 (4)	0.0039 (4)	0.0021 (4)
O2	0.0110 (5)	0.0107 (5)	0.0115 (5)	−0.0028 (4)	0.0083 (4)	−0.0020 (4)
O3	0.0076 (5)	0.0097 (5)	0.0130 (5)	−0.0010 (4)	0.0061 (4)	−0.0028 (4)

### Hexaaquadichloridolutetium(III) chloride (Lu) Geometric parameters (Å, º)

**Table:**

Lu1—Cl1^i^	2.7113 (4)	Lu1—O3^i^	2.2917 (12)
Lu1—Cl1	2.7113 (4)	O1—H1A	0.77 (3)
Lu1—O1^i^	2.3246 (11)	O1—H1B	0.76 (3)
Lu1—O1	2.3246 (11)	O2—H2A	0.73 (3)
Lu1—O2^i^	2.3272 (12)	O2—H2B	0.82 (3)
Lu1—O2	2.3272 (12)	O3—H3A	0.79 (3)
Lu1—O3	2.2917 (12)	O3—H3B	0.77 (3)
			
Cl1—Lu1—Cl1^i^	83.270 (15)	O3^i^—Lu1—O1	69.54 (4)
O1—Lu1—Cl1^i^	146.14 (3)	O3—Lu1—O1	76.23 (4)
O1^i^—Lu1—Cl1	146.14 (3)	O3^i^—Lu1—O1^i^	76.23 (4)
O1^i^—Lu1—Cl1^i^	76.23 (3)	O3—Lu1—O1^i^	69.53 (4)
O1—Lu1—Cl1	76.23 (3)	O3—Lu1—O2	70.87 (4)
O1^i^—Lu1—O1	133.30 (6)	O3^i^—Lu1—O2^i^	70.87 (4)
O1—Lu1—O2	72.85 (4)	O3^i^—Lu1—O2	138.80 (4)
O1—Lu1—O2^i^	121.47 (4)	O3—Lu1—O2^i^	138.80 (4)
O1^i^—Lu1—O2	121.47 (4)	O3^i^—Lu1—O3	84.31 (6)
O1^i^—Lu1—O2^i^	72.85 (4)	Lu1—O1—H1A	118 (2)
O2—Lu1—Cl1	78.41 (3)	Lu1—O1—H1B	125.0 (19)
O2^i^—Lu1—Cl1^i^	78.41 (3)	H1A—O1—H1B	106 (3)
O2—Lu1—Cl1^i^	76.86 (3)	Lu1—O2—H2A	119 (2)
O2^i^—Lu1—Cl1	76.86 (3)	Lu1—O2—H2B	121.7 (19)
O2—Lu1—O2^i^	146.71 (6)	H2A—O2—H2B	107 (3)
O3—Lu1—Cl1	143.49 (3)	Lu1—O3—H3A	118 (2)
O3—Lu1—Cl1^i^	107.72 (3)	Lu1—O3—H3B	117.7 (19)
O3^i^—Lu1—Cl1	107.72 (3)	H3A—O3—H3B	109 (3)
O3^i^—Lu1—Cl1^i^	143.49 (3)		

Symmetry code: (i) −*x*+1, *y*, −*z*+1/2.

### Hexaaquadichloridolutetium(III) chloride (Lu) Hydrogen-bond geometry (Å, º)

**Table:**

*D*—H···*A*	*D*—H	H···*A*	*D*···*A*	*D*—H···*A*
O1—H1*A*···Cl1^ii^	0.77 (3)	2.42 (3)	3.1591 (13)	163 (3)
O1—H1*B*···Cl2^iii^	0.76 (3)	2.40 (3)	3.1625 (12)	171 (2)
O2—H2*A*···Cl1^iv^	0.73 (3)	2.42 (3)	3.1517 (13)	175 (3)
O2—H2*B*···Cl2^v^	0.82 (3)	2.45 (3)	3.2327 (13)	160 (3)
O3—H3*A*···Cl1^vi^	0.79 (3)	2.34 (3)	3.1290 (13)	174 (3)
O3—H3*B*···Cl2	0.77 (3)	2.41 (3)	3.1653 (12)	167 (3)

Symmetry codes: (ii) −*x*, −*y*, −*z*; (iii) −*x*, −*y*+1, −*z*; (iv) *x*, −*y*, *z*+1/2; (v) *x*, *y*−1, *z*; (vi) −*x*+1, *y*+1, −*z*+1/2.
